# Identification and Validation of the ESR1 Y537S Mutant–Enriched Enhancer and Its Downstream Target IGFBP3

**DOI:** 10.1158/2767-9764.CRC-26-0366

**Published:** 2026-10-14

**Authors:** Yonatan Amzaleg, Desirea Mecenas, Ebony Flowers, Lin Li, Amal Thomas, Haley Hendrick, Hyeonguk Noh, Aidan Moriarty, Yiru Wang, Zheqi Li, Yuji Zhang, Remi Klotz, Steffi Oesterreich, Min Yu

**Affiliations:** 1 Department of Stem Cell Biology and Regenerative Medicine, Keck School of Medicine, University of Southern California, Los Angeles, California.; 2 USC Norris Comprehensive Cancer Center, Keck School of Medicine, University of Southern California, Los Angeles, California.; 3 Center for Craniofacial Molecular Biology, Ostrow School of Dentistry, University of Southern California, Los Angeles, California.; 4 Department of Molecular and Computational Biology, USC David and Dana Dornsife College of Letters, Arts and Sciences, University of Southern California, Los Angeles, California.; 5 Department of Pharmacology and Physiology, University of Maryland School of Medicine, Baltimore, Maryland.; 6 Marlene and Stewart Greenebaum Comprehensive Cancer Center, University of Maryland School of Medicine, Baltimore, Maryland.; 7 Department of Pharmacology and Chemical Biology, University of Pittsburgh School of Medicine, Pittsburgh, Pennsylvania.; 8 Department of Epidemiology and Public Health, University of Maryland School of Medicine, Baltimore, Maryland.

## Abstract

Endocrine therapy that inhibits estrogen receptor (ER)–mediated signaling is a cornerstone treatment for ER-positive breast cancer, a subtype that accounts for more than 75% of all breast cancer cases. However, resistance to endocrine therapy occurs in a significant proportion of patients, and hotspot missense point mutations of the *ESR1* gene that codes for ERα are one of the major underlying mechanisms for such resistance. Although recent research has provided novel insights into the functions and activities of mutant ER, effective therapeutics that can overcome this mutant ER-mediated endocrine resistance remain elusive. Using a circulating tumor cell line derived from a patient with metastatic breast cancer and harboring the *ESR1* Y537S mutation, together with genetically engineered breast cancer cell lines carrying the same mutation, we identified a novel enhancer region that is preferentially occupied by Y537S mutant ER. Deletion of this enhancer region reduced cell proliferation and adhesion *in vitro*, decreased tumorigenicity *in vivo*, and enhanced sensitivity to fulvestrant specifically in cells harboring the Y537S ER mutation. We subsequently identified insulin-like growth factor–binding protein 3 as a functional downstream target of this enhancer and validated its contribution to a subset of the phenotypes associated with the Y537S mutant ER.

**Significance:**

Mutations in the ER can drive treatment resistance in advanced breast cancer. We identify a previously unrecognized DNA regulatory region that supports growth and drug resistance in tumors carrying the common Y537S mutation, revealing a new mechanism of resistance and a potential therapeutic vulnerability.

## Introduction

Breast cancer is the most common cancer and the second leading cause of cancer-related deaths in women (1). As approximately 80% of breast cancers express estrogen receptor alpha (ERα; ref. 2), targeting ER signaling through endocrine therapies remains important for treating patients with ER-positive breast cancer (3). The type of endocrine therapies administered to patients depends on the source of estrogen. For premenopausal women, the endocrine therapy includes selective estrogen receptor modulators, which compete with estrogen to bind ERα, thereby preventing ERα activation, or selective estrogen receptor degraders (SERD), which inhibit ER-mediated functions by promoting ER degradation (4). For postmenopausal women, aromatase inhibitors are commonly used to block the aromatase enzyme (CYP19A1), which normally converts androgens to estrogens in the peripheral tissues (5).

In early-stage breast cancer, endocrine therapy reduces the recurrence rate by 50% and reduces the mortality rate by more than 25% (6). However, approximately 30% of patients with ER-positive breast cancer are innately resistant to endocrine therapy, and 40% of patients who initially responded to therapy develop resistance with continued treatment (7–9). Studies performing exome sequencing on endocrine therapy–resistant metastatic tumor samples identified several frequent point mutations in the ERα protein-encoding gene, *ESR1*, with most mutations found in the ligand-binding domain (10). *In vitro* studies have shown that these single point mutations lead to conformational changes that mimic the ligand-activated form of ERα in the absence of an agonist (11–13). These mutations occur in 18% to 53% of patients with breast cancer that has advanced while on endocrine therapy (11–13), suggesting a role in developing resistance. Data from molecular dynamics modeling (11, 14) illustrate an attenuated affinity for ER antagonists in cells harboring the two most common ER mutations, Y537S and D538G. *In vitro* data from our own circulating tumor cell (CTC) line harboring the Y537S mutation (15), as well as ERα-positive cell lines that were edited to harbor the *ESR1* mutation, exhibit a decreased effect of the SERD fulvestrant (ICI182780) on cell proliferation (10, 16). Therefore, identifying the mutant ERα-specific mechanism of function may inform new strategies to overcome the mutant ERα-mediated resistance to endocrine therapies.

RNA sequencing (RNA-seq) and chromatin immunoprecipitation sequencing (ChIP-seq) data from various labs employing ERα-positive cell lines genetically modified to harbor *ESR1* mutations have shown that in addition to expressing traditional ERα target genes and binding to traditional ERα binding sites, ERα mutants exhibit distinct transcriptomes and binding landscapes compared with the activated wild-type (WT) ERα (17–20). By analyzing these data along with ERα ChIP-seq results from our Y537S mutant ERα CTC line, BRx68 (15), we identified an intronic region of the pseudogene *DPY19L1P1* on chromosome 7 as an enhancer in which Y537S ERα binds with higher intensity compared with the activated WT ERα, followed by phenotypic validation and identification of insulin-like growth factor–binding protein 3 (*IGFBP3*) as a potential downstream gene.

## Materials and Methods

### Cell culture

The ER^+^ CTC cell line BRx68 was generated from a previous study (15) and was cultured in ultralow attachment plates with 1× RPMI 1640 media supplemented with EGF (20 ng/mL), basic fibroblast growth factor (20 ng/mL), 1× B27, and 1× antibiotic/antimycotic, in 4% O_2_ and 5% CO_2_.

Genetically edited MCF7 cells (WT and those bearing the Y537S mutation) were obtained from Dr. Ben Ho Park’s lab and maintained in high-glucose DMEM supplemented with 10% FBS and 1% penicillin–streptomycin (P/S). For assays that involve treatment with estradiol (E2; Sigma-Aldrich, E2758) and fulvestrant (ICI 182780; Sigma-Aldrich, I4409), cells were washed twice with PBS and then grown in phenol red–free high-glucose DMEM supplemented with 10% charcoal/dextran-stripped fetal bovine serum (CSS; GeminiBio, A76G02J), 1% L-glutamine (L-Q; Sigma-Aldrich, G3126), and 1% P/S for a period of at least 3 days. The cells were then detached using TrypLE Express Enzyme (Thermo Fisher Scientific, 12604013) at 37°C for 5 minutes and plated for the respective assays. T47D cells with the respective genotypes WT and Y537S were also obtained from Dr. Steffi Oesterrich’s lab and maintained in RPMI media supplemented with 10% FBS and 1% P/S. For assays in which cells needed to be grown in a hormone-deprived state, cells were washed twice with PBS and then maintained in RPMI media supplemented with 10% CSS, 1% L-Q, and 1% P/S for 3 days before the cells were detached using TrypLE Express Enzyme and then plated for their respective assays.

Cell line authentication was evaluated based on short tandem repeat loci analysis using the GenePrint 24 system (Promega) by the Translational Genomics Laboratory of the University of Maryland School of Medicine. The last authentication was performed on July 23, 2026. Routine testing for mycoplasma contamination was conducted monthly using the e-Myco PLUS Mycoplasma PCR Detection Kit (Bulldog-Bio). The last mycoplasma testing was performed on July 22, 2026. The number of passages between thawing and the described experiments was within 10 to 12 passages.

### ChIP-seq

*Ex vivo* BRx68 cells were grown in suspension at a seeding density of 10 million cells in a 500 cm^2^ Thermo Fisher Scientific NUNC Square Culture dish. The experiment was performed in two biological replicates and consisted of two conditions: BRx68 cells treated with a final concentration of 10 nmol/L of E2 and the respective vehicle, ethanol. Following 24 hours of growth with the drug or vehicle, cells were collected and processed according to an optimized protocol developed by the Peggy Farnham lab (21). Cells were collected, centrifuged, and resuspended in 1% formaldehyde for 8 minutes while on a shaker to cross-link. Cross-linking was stopped by adding glycine at a final concentration of 0.125 mol/L. The cross-linked cell solution was then centrifuged at 430 rcf for 5 minutes at 4°C and washed twice with ice-cold PBS. To lyse cells and extract the nuclei, the pellets were resuspended in cell lysis buffer [5 mmol/L piperazine-N,N′-bis(2-ethanesulfonic acid; pH 8), 85 mmol/L KCl, 1% IGEPAL, and protease inhibitors: 1 mmol/L phenylmethylsulfonyl fluoride, 1 μg/mL aprotinin, and 1 μg/mL leupeptin] and incubated on ice for 15 minutes. The cells were then centrifuged at 430 rcf for 5 minutes and decanted, and the pellet was resuspended in nuclear extraction buffer. Following a 30-minute incubation on ice, the samples were sonicated using a BioRuptor (high setting with cycles of 30 seconds on and off for 15 minutes). Sonication was verified by running the samples on an agarose gel. Ten micrograms of the sonicated chromatin was then incubated with either ER antibody (Santa Cruz Biotechnology, HC-20) or IgG (Cell Signaling Technology, #2729) corresponding to the species of the primary antibody (rabbit) overnight. The following day, the chromatin–antibody incubated samples were resuspended with ChIP-grade Protein A/G beads for 3 hours, washed three times with immunoprecipitation buffers, and then de-cross-linked overnight. The samples were then column purified with the Zymo DNA concentration kit and made into libraries via the KAPA HyperPrep Kit for next-generation sequencing at 15 million reads per sample.

### Data processing and analysis of ChIP-seq data from BRx68

Single-end FASTQ files of each sample underwent a quality check using FastQC v0.11.9 (https://www.bioinformatics.babraham.ac.uk/projects/fastqc/). The files were then trimmed of their adaptors using Cutadapt v3.4 (released March 30, 2021) tool (22) at settings optimized for ChIP-seq. The samples were then aligned to the hg19 genome build using Bowtie v1.3.0 (code: bowtie -q -p 8 -m 1 -v 3 –sam best; ref. 23), converted to BAM files and sorted via Samtools v1.11 (24), and deduplicated using the MarkDuplicates.pl tool from Picard v2.25.0. To generate heatmaps, the bamCoverage tool from the deepTools v3.5.0 package (25) was used to generate bigwig files normalized using reads per genomic content, which takes into consideration genome size and the total number of reads. Once the bigwig files were generated, the computeMatrix tool from the deepTools v3.5.0 package (25) was used to generate an intermediary file of all the scores of each sample’s bigwig file against certain genomic regions. For purposes of determining the putative WT ER and mutant Y537S ER binding sites, publicly available ChIP-seq datasets from the Myles Brown lab (17) were used, calling statistically significant peaks in the same way as in the case of the BRx68 samples WT ER binding sites. The MACS2 v2.2.7.1 tool (26) was used to call peaks showing statistical significance over input DNA in MCF7 WT cells in the presence of E2. For the Y537S mutant ER-specific peaks, the peaks for MCF7 Y537S cells were called, and the bedtools (Bedtools v2.30.0) -v function (27) was used to remove peaks also called in the WT ER samples. The plotHeatmap tool from the deepTools v3.5.0 (25) package was then used to graphically display the scores of each sample against those regions.

For purposes of the principal component analysis (PCA) plot, a count table was generated using featureCounts (Subread) v2.0.1 (28). In short, an SAF file was first generated following the calling of significant peaks in all BRx68 samples, and the bedtools intersect -wa -wb function was used to combine the list of significant genomic coordinates. This was followed by using the -uniq command to remove peaks that were duplicated. The featureCount tool was used with the code featureCounts -a ${saf_file} -F SAF –readExtension3 150 -o count_table.txt -O -T 8 to determine a numeric count for each sample against all possible genomic regions. The PCA plot was then plotted against the top 1,000 variable regions across all samples and visualized using ggplot2 v3.3.5.

The feature distribution chart was generated using the ChIPseeker v1.30.3 package (29). Statistically significant called peaks were piped into the R program, and the promoter was identified as being 1 kb upstream and downstream of the transcription start site (TSS).

### Data analysis of publicly available datasets for enhancer discovery

Our BRx68 ER ChIP-seq dataset was analyzed along with ER ChIP-seq datasets from the labs of Miles Brown (17), Simak Ali (19), and Steffi Oesterrich (20). The processing of the files was performed using the same pipeline employed for the BRx68 FASTQ files. For the purposes of determining significant peaks, the processing varied based on the datasets. To determine mutant-specific peaks in the datasets that included only one sample per condition (i.e., no replicates), the MACS2 v2.2.7.1 callpeak function (26) was called on all processed BAM files with the same parameters against the respective input control (sonicated genomic DNA without an antibody). Sample code is as follows: macs2 callpeak -t $(sample.bam) -c $(control_Input.bam) -f BAM -g hs -n sample_peaks -q 0.01. Significant peaks were called for mutant and WT ER samples, and then bedtools intersect -v was used to remove from the called mutant ER peaks those that were also found in the WT ER. The mutant ER–unique peaks were then compared against the BRx68 called peaks to identify shared peaks across the study. For the ER ChIP-seq with replicates (17), mutant-specific ER peaks were determined via the featureCount (28) tool, as with the BRx68 samples. In this case, the SAF file featured all possible ER binding regions across all samples. The count table was then piped into DESeq2 v1.30.1 for differential binding analysis, with parameters set at mutant ER log_2_ fold change greater than 1 compared with WT ER, with a false discovery rate (FDR) value of less than 0.05. This list was then compared with the BRx68 list to produce a list of common mutant ER–specific binding peaks across the samples. The shared regions were then visualized on the UCSC Genome Browser (30) to determine the levels of three different epigenetic marks associated with regulatory elements—monomethylation of lysine 4 on histone H3 protein subunit (H3K4me1), trimethylation of lysine 4 on histone H3 protein subunit (H3K4me3), and acetylation of lysine 27 on histone H3 protein subunit (H3K27Ac)—across seven different cell lines.

### RNA-seq and data processing

MCF7 WT and Y537S cells harboring the DPY191L1 region deletion (WT_DPY_KO and Y537S_DPY_KO, respectively) along with their respective controls (WT_C10, WT_C11, Y537S_D4, Y537S_Z99) were seeded in a 24-well plate at a seeding density of 25,000 cells per well for 2 days prior to RNA extraction. Given that only one clone for either genotype of MCF7 cells that contained the DPY region deletion was able to be isolated, we performed biological replicates of the same clones for RNA-seq. For controls, we used two different clones for each genotype as biological replicates. After 2 days in culture, RNA was extracted using the Zymo Quick RNA kit according to the manufacturer’s instructions. Extracted, high-quality RNA was sent to Novogene Corporation for paired-end sequencing at 15 million reads per sample. Paired-end FASTQ files were quality-checked using FastQC v0.11.9 (31). The reads were then mapped to the human genome build GRCh37 from Ensembl (https://ftp.ensembl.org/pub/grch37/current/fasta/homo_sapiens/dna/Homo_sapiens.GRCh37.dna_sm.primary_assembly.fa.gz) using STAR v2.7.9a (32). The aligned reads were then counted via featureCounts (Subread) v2.0.1 (28) and piped into the R program DESeq2 v1.30.1 (33) for normalization to sequencing depth for downstream analysis. For the purposes of producing the PCA plot, the count table was transformed using the vst function under the standard parameters recommended by the developers of DESeq2 v1.30.1 (33). The transformation was done to eliminate the experiment-wide trend of variance over mean. The PCA plot was then generated using ggplot2 v3.3.5 (34). DeepTools v3.5.0 (25) was used to generate bigwig files to visualize using the WashU Epigenome Browser (35) and the UCSC Genome Browser (30). For purposes of differential expression analyses, the contrast function was used, and with each comparison, we looked at differentially expressed genes between two samples to have an FDR value of less than 0.05 and a log_2_ fold change value greater than 0. The R Venn.diagram package was used to visually depict shared upregulated genes across the different comparisons. We used the intersect function in R to identify shared upregulated genes between two or more conditions and the setdiff function to determine which genes are upregulated in certain comparisons and not others.

RNA-seq analysis of publicly available datasets (17–19) involved the same steps as mentioned above to ensure consistency when analyzing the data. To determine transcriptomic differences between the constitutively active Y537S mutant ER and the E2-activated WT ER, the differential expression analysis in all of the studies had to take into consideration that there are genes that may be upregulated because of the vehicle treatment in the Y537S cells (ethanol) over the E2 treatment in WT cells. To correct for this, for each study, we performed the following set of comparisons: For all comparisons, we used the parameters of log_2_ fold change greater than 1 and an FDR value of less than 0.05. A standard comparison was first performed, determining the upregulated genes in Y537S cells in the vehicle setting (Y537S_veh) compared with WT cells in the presence of E2 (WT_E2). We then identified upregulated genes in WT_veh cells compared with WT_E2 cells. After generating the lists, genes that were statistically significantly upregulated in WT_veh over WT_E2 were removed from the Y537S_veh versus WT_E2 list. This was then intersected with the Y537S_E2 versus WT_veh list to develop a comprehensive list of Y537-upregulated genes compared with the activated WT.

For the MET500 cohort analysis, RNA-seq FASTQ files from 91 metastatic breast cancer samples were obtained from the Database of Genotypes and Phenotypes (accession phs000673.v2.p1; ref. 36). Transcript-level quantification was performed using Salmon (v0.8.2), and transcript counts were summarized to gene-level counts with tximport. Gene-level counts from all samples were jointly normalized using the trimmed mean of M values (TMM) method implemented in edgeR. Log_2_-transformed TMM-normalized counts per million were used for downstream analyses. A total of 46 ER-positive (ER^+^) samples were selected and included in the final analysis, consistent with our previous report (37). ESR1 mutation status was obtained from the MET500 portal (https://met500.path.med.umich.edu).

### Chromatin accessibility profiling via Assay for Transposase-Accessible Chromatin with high-throughput sequencing and data processing

Assay for Transposase-Accessible Chromatin with high-throughput sequencing (ATAC-seq) was performed as previously described (38). In short, nuclei preparation was performed by resuspending 50,000 MCF7 WT, Y537S, and D538G cells in ATAC-seq lysis buffer [10 mmol/L Tris (pH 7.4), 10 mmol/L NaCl, 3 mmol/L MgCl_2_, 0.1% IGEPAL]. The transposition reaction was performed using the Tn5 transposase (Nextera) at 37°C for 30 minutes with mild shaking. Transposed DNA was amplified by PCR using 100× SYBR I Green (similar to traditional SYBR used for quantitative real-time PCR (qPCR or qRT-PCR), but lacking any dNTPs and polymerase enzyme) for 20 cycles of labeling. The amplified PCR product was then run on a CFX iCycler real-time PCR machine (Bio-Rad). The R value, which corresponds to the cycle value in which the relative fluorescent unit of a certain sample reaches its saturation point, was calculated for each individual sample and divided by 3 to determine the number of additional cycles necessary for each. The generated libraries were then purified using Agencourt AMPure XP (Beckman Coulter). Library quality was determined using TapeStation, and the prepared libraries were sent to Novogene for paired-end sequencing at 10 million reads per sample. The resulting FASTQ files were processed in the same way as detailed for the ChIP-seq FASTQs. The “differential open” regions were determined using featureCounts (28) to produce a count table similar to the way we performed the differential binding analysis with the BRx68 ChIP-seq samples. The count table was then piped into DESeq2 (33) to perform the different comparisons using the contrast function, using a log_2_ fold change value of greater than 1 and an FDR value of less than 0.05. The lists of regions were then annotated using the R program biomaRt (39), and the resultant gene lists were then piped into integrative pathway analysis technology (40) to determine enriched pathways.

### CRISPR-Cas9 to knockout genomic regions

To generate the deletion of the DPY19L1P1 region, single-guide RNAs (sgRNA) corresponding to 200 to 300 bp upstream of the leftmost part of the region (L-sgRNA) and 200 to 300 bp downstream of the rightmost part (R-sgRNA) were designed using *CRISPOR* (41). The L-sgRNA was cloned into the backbone vector called pSpCas9(BB)-2A-GFP (henceforth called px458) that expresses GFP. The R-sgRNA was cloned into another backbone vector called pLKO5.sgRNA.EFS.tRFP that expresses RFP. Both cloning of the L-sgRNA and R-sgRNA were done according to the published protocol previously (42). MCF7 WT and Y537S cells were seeded at a density of 300,000 cells per well in a six-well plate. Px458 containing L-sgRNA and pLKO5.sgRNA.EFS.tRFP containing R-sgRNA were transfected into both cell genotypes using Lipofectamine 3000 (Thermo Fisher Scientific) according to the manufacturer’s optimized protocol. Following 2 days of transfection, cells were washed, trypsinized, resuspended in 1% BSA media, and FACS-sorted based on positivity for both RFP and GFP and negativity for 4′,6-diamidino-2-phenylindole (DAPI) into single cells per well in a 96-well plate. The individual wells were observed for 2.5 weeks until viable colonies were identified. To identify clones harboring a knockout (KO) of the DPY region, genomic DNA was extracted from different clones of both cell genotypes, and a Taq polymerase–based PCR was performed using primers that were 300 bp upstream and downstream of the approximately 500 bp DPY region. PCR products were run on a 1% agarose gel that was supplemented with GreenGlo Safe DNA Dye (Denville Scientific, CA3600) and visualized using the Bio-Rad Gel EQ and the Quantity One analyzing software.

IGFBP3 KO cells were produced by cloning in sgRNAs (Sg1.1, Sg2.1) targeting different regions within the first exon of IGFBP3 into the px458 vector. Following 2 days of transfection, cells were washed, trypsinized, resuspended in 1% BSA media, and single cell–sorted for GFP-positive and DAPI-negative cells. The single cells were observed under a microscope regularly for growth, with media changes taking place following the first week of sorting. When single-cell clones developed, secretion of IGFBP3 in the extracellular media was subjected to an ELISA to confirm the successful KO clones.

### Cell proliferation assay with CellTiter-Glo

MCF7 WT and Y537S cells harboring the DPY19L1P1 deletion (WT_DPY_KO and Y537S_DPY_KO, respectively) and their respective controls were seeded at a density of 750 cells per well in technical triplicates on a 96-well plate. The cells were grown for 0, 3, or 7 days. To determine the proliferation change of the cells at the different time points, cells were lysed with CellTiter-Glo 2.0 (CTG) reagent (Promega, G924) at a final concentration of 1:2 of reagent to culture media. The entire mix was then transferred to a flat-bottom, white plate (CoStar, cat. #3912), and the luminescence was read using the CLARIOstar microplate reader. The values were normalized to the seeding control (day 0). This assay was repeated 2 more times for a total of three biological replicates. For the IGFBP3 KO proliferation assay, the same setup was used as above, with a starting seeding density of 750 cells per well and two time points recorded (days 0 and 7).

For the purposes of measuring cell growth in the presence of different concentrations of ICI with and without E2, WT_DPY_KO and Y537S_DPY_KO cells (along with their respective controls) were seeded at 750 cells per well in a 96-well plate in CSS media. The following day, the CSS media was removed and replaced with CSS media supplemented with differing concentrations of ICI (ranging from 1–1,000 nmol/L) and/or E2 (1 nmol/L). The cells were also treated with the respective vehicles of ICI and E2 as the controls. The media changes were done on the fourth day after seeding (the third day following the first treatment), and the cells were lysed with CTG, and the luminescence was read using the CLARIOstar microplate reader. The values were normalized to the seeding control (day 0). This experiment was performed for a total of six biological replicates.

The experiment above was also performed for the MCF7 Y537S IGFBP3 KO cells but treated with a final concentration of either 10 or 100 nmol/L ICI. This experiment was performed for three biological replicates.

### Long-term ICI treatment assay

To determine the effect of long-term ICI treatment on MCF7 DPY19L1P1 deletion cells and their respective controls, 75,000 cells were seeded in a 12-well plate in regular medium with complete serum supplemented with either 10 nmol/L of ICI or its vehicle (DMSO). After 3 days, cells were washed, trypsinized, and counted to determine proliferation. A total of 75,000 cells from this first proliferation were seeded again, along with their respective treatment. This process was repeated over the course of 3 weeks, with cells trypsinized and counted every 3 days. We then multiplied all the fold changes from each of the 3-day checkpoints, and the final multiplier of the vehicle-treated cells was divided by the multiplier of the respective ICI-treated cells. This experiment was performed for a total of four biological replicates.

### Proliferation assay with crystal violet

WT_DPY_KO and Y537S_DPY_KO cells were grown in CSS media for 3 days and dissociated by incubating with TrypLE express enzyme (Thermo Fisher Scientific, cat. #12604013) at 37°C for 5 minutes. Cells were plated at a density of 4,000 cells/well in 1 mL volume per well in 24-well tissue culture-treated plates. The following day, the media was replaced with CSS media supplemented with vehicle (0.1% EtOH and 0.1% DMSO), 1 nmol/L of E2, 100 nmol/L of ICI, and 1 nmol/L of E2 + 100 nmol/L of ICI. The media changes were done on the fourth day after seeding (the third day following the first treatment). At the end of treatment, cells were washed twice with cold PBS containing 1 mmol/L CaCl_2_ and 1 mmol/L MgCl_2_. Cells were then fixed with cold 100% methanol for 10 minutes at room temperature, followed by washing with PBS three times. Crystal violet (0.5% w/v crystal violet in 20% ethanol) was added to the cells at 150 μL per well and incubated on a shaker for 10 minutes at room temperature. The plates were then submerged in large 1 L beakers of distilled water for 1 minute each with gentle twisting. Plates were then dried overnight in a fume hood and imaged the following day using a Keyence BZ-X810 microscope at a 4× magnification. Crystal violet was then recovered by adding 200 μL of 100% methanol to the wells and then placed to shake for 15 minutes at room temperature. The recovered crystal violet solution (100 μL) was transferred to flat-bottom 96-well plates (Corning, cat. #9017), and absorbance was measured at 590 nm using a BioTek plate reader.

### Cell adhesion assay with crystal violet

Rat tail collagen I (Corning, 354236) was diluted in 0.2N acetic acid to a final concentration of 100 μg/mL and was used to coat the wells of a 24-well plate overnight at 37°C. The following day, the collagen was removed and washed twice with PBS. The collagen-coated wells were then blocked in DMEM with 10% FBS and incubated for 1 hour at 37°C. During this time, cells were trypsinized, counted, and adjusted to an optimized final seeding density (30,000 cells per well) and adhered to the collagen-coated wells for 30 minutes at 37°C. The plate was then inverted and washed twice with ice-cold PBS supplemented with CaCl_2_ and MgCl_2_ to a final concentration of 1 mmol/L to remove the unadhered cells. Cells were then fixed with cold 100% methanol for 10 minutes at room temperature, followed by washing with PBS 3 times. Crystal violet (0.5% w/v crystal violet in 20% ethanol) was added to the cells at 150 μL per well and incubated on a shaker for 10 minutes at room temperature. Following this, the plates were submerged in large 1 L beakers of distilled water for 1 minute each with gentle twisting. The plates were then dried overnight in a fume hood and imaged the following day using a Keyence BZ-X810 microscope at 4× magnification. Crystal violet was then recovered by adding 200 μL of 100% methanol to the wells and placed to shake for 15 minutes at room temperature. The recovered crystal violet solution (100 μL) was transferred to flat-bottom 96-well plates (Corning, REF 9017), and absorbance was measured at 590 nm using a BioTek plate reader. This experiment was performed the same for the DPY191L1 region deleted cells and their controls, as well as the IGFBP3 KO cells and their controls.

### Methylcellulose growth assay

To determine the effect of suspension conditions, GFP-positive Y537S MCF7 cells with SgC or DPY KO cells with empty plasmid control or rescued with IGFBP3 overexpression were seeded at a density of 1,000 GFP-positive cells per well in 12-well ultralow attachment plates. The cells were suspended in 2 mL of regular growth medium supplemented with 1.5% methylcellulose. To monitor growth and survival, the plates were imaged over a period of 7 days using the LICORbio’s Odyssey M imaging system on the 488 nm channel. The number of resulting colonies was subsequently quantified using ImageJ Fiji software. To determine the proliferation change, the fold change in colony number was calculated and normalized to the initial day 1 quantification.

### ELISA

Cells were incubated with insulin for 24 hours at 37°C. All ELISA reagents from the human IGFBP3 ELISA kit (Invitrogen, EHIGFBP3) were allowed to come to ambient temperature before use, per protocol recommendation. Media (100 μL) from each well of the 24-well plate was loaded into three wells of the provided IGFBP3 antibody-coated 96-well plate. A provided IGFBP3 standard was diluted to points 18,000, 6,000, 2,000, 666.67, 222.22, and 74.07 pg/mL and was also plated into each well of the antibody-treated plate, as well as a provided blanking reagent. The plate was sealed and incubated at 4°C overnight on a shaking platform. The antibody-coated plate was treated with a biotinylated detection antibody, a streptavidin–HRP, a chromogen substrate, and a reaction stop solution according to kit instructions. After the stop solution is added, the absorbance is read at 450 and 540 nm, and the 540 nm values are subtracted from the 450 nm values to correct for any background signal. The concentration of IGFBP3 in the cell supernatant samples can then be calculated using the standard curve. This assay was repeated 2 more times for a total of three biological replicates.

### qRT-PCR

RNA was extracted using the Quick RNA Microprep kit from Zymo. RNA (500 ng) was reverse transcribed using 5× iScript supermix (Bio-Rad). The cDNA was diluted to 5 ng/μL, and 2 μL of cDNA was used per qRT-PCR using iQ SYBR Green Supermix (Bio-Rad). All reactions were run on a CFX iCycler real-time PCR machine (Bio-Rad). The primer list can be found in a comprehensive list of all primers and sgRNA sequences used.

### Cleavage under targets and tagmentation

Cleavage under targets and tagmentation (CUT&Tag) was performed using an optimized protocol from EpiCypher CUTANATM (43). Briefly, MCF7 WT and Y537S were seeded at a density of 25,000 cells per well in a 24-well plate and extracted the following day via trypsinization. Cells were resuspended in 100 μL of wash buffer and 10 μL of prewashed magnetic concanavalin A (conc-A) beads, which were incubated with the cells for 15 minutes. The conc-A beads are important for tethering the cells, thereby increasing the efficiency of the assay. The supernatant was then removed and resuspended with antibody buffer containing 1 μg of rabbit polyclonal H3K4me1 from Abcam (ab8895), recommended by EpiCypher for the assay, and allowed to incubate in a cold room overnight under shaking conditions. The following day, the supernatants were removed, and the beads were resuspended with digitonin-based wash buffer supplemented with 1 µg of rabbit secondary from Cell Signaling Technology (2729). This was incubated for 1 hour at room temperature. The cells were then washed 3 times with digitonin buffer, and on the third wash, digitonin buffer was supplemented with a Tn5 conjugated to Protein A/G (supplied by EpiCypher) and incubated for 1 hour at room temperature. This was then washed 3 times afterward with digitonin buffer and resuspended in tagmentation buffer (digitonin-based wash buffer with the addition of MgCl_2_ at a final concentration of 10 mmol/L to activate the tagmentation) and incubated at room temperature for 10 minutes. The tagmentation was then quenched by using an SDS-based quenching buffer. As CUT&Tag uses similar principles to ATAC-seq, the library prep method for CUT&Tag is the same. The libraries were then sent to Novogene for paired-end read sequencing at 10 million reads per sample. Data processing followed the same methods used to analyze the ChIP-seq datasets.

### List of primers

#### Primer sequences for qRT-PCR

*GREB1*: -F 5′-ATG​GGA​AAT​TCT​TCG​CTG​GAC-3′; -R 5′-CAC​TCG​GCT​ACC​ACC​TTC​T-3′
*IGFBP3*: #1 -F 5′-ATC​TGT​GCT​CTG​CTG​AGA​CTC​GT-3′; -R 5′-CCA​CCC​CCT​CCA​TTC​AAA​GAT-3′
*IGFBP3*: #2 -F 5′-GAG​CCT​GAC​TTT​GCC​AGA​CC-3′; -R 5′-CTG​GGA​AAG​GAG​CTC​ACG​C-3′
*CD44*: -F 5′-TGG​TCG​CTA​CAG​CAT​CCT​CTC-3′; -R 5′-TGT​GGG​CAA​GGT​GCT​ATT​GA-3′
*36B4*: -F 5′-GTG​TTC​GAC​AAT​GGC​AGC​AT-3′; -R 5′-AGA​CAC​TGG​CAA​CAT​TGC​GGA-3′

#### Primer sequences for ChIP-qPCR

*GREB1*: -F 5′-ACC​GCA​ACG​TGG​TTT​TCT​CAC​CCT​ATG​G-3′; -R 5′-GAG​GTA​GGA​CCA​GCA​ATC​CAT-3′
*DPY19L1P1* region: -F 5′-GAA​GAA​CCA​AAC​TGC​ACG​ACG​AAA-3′; - R 5′-GAG​GTA​GGA​CCA​GCA​ATC​CAT-3′

#### Primer sequences for cloning

##### DPY19L1P1 KO

L-sgRNA: -F 5′ caccg TGGGACGTTAAGGTGTTGAT 3′; -R 5′ aaac ATCAACACCTTAACGTCCCAc 3′
R-sgRNA: -F 5′ caccg CGTGCAGTTTGGTTCTTCAC 3′; -R 5′ aaac GTGAAGAACCAAACTGCACGc 3′

##### IGFBP3 KO

L-sgRNA 1.1: -F 5′ caccg CTGACGTGCGCACTGAGCGA 3′; -R 5′ aaac TCGCTCAGTGCGCACGTCAGc 3′
L-sgRNA 2.1: -F 5′ caccg CACCAGCTCCGCGCACACGG 3′; -R 5′ aaac CCGTGTGCGCGGAGCTGGTGc 3′

#### Primer sequence for PCR

DPY19L1P1: -F 5′ ACC​AGT​AGC​TGC​CAA​AGT​TTA 3′; -R 5′ TGAACA​TCA​ACA​TCC​AGT​CAT​T 3′

### *In vivo* experiment

Animal experiments were performed in accordance with protocols approved by Institutional Animal Care and Use Committees at the University of Southern California (#20431). MCF7s were cultured for 3 days in hormone-free CSS media. Cells [100 μL (1 × 10^5^)] in PBS and Matrigel (Corning) were injected into the fourth mammary fat pad of 6- to 8-week-old ovariectomized female NOD/SCID gamma (NSG) mice (The Jackson Laboratory, Strain #005557). Tumor growth was monitored by caliper.

## Results

### ER binding profile of a CTC line harboring the heterozygous Y537S ER mutant

**Figure 1.**
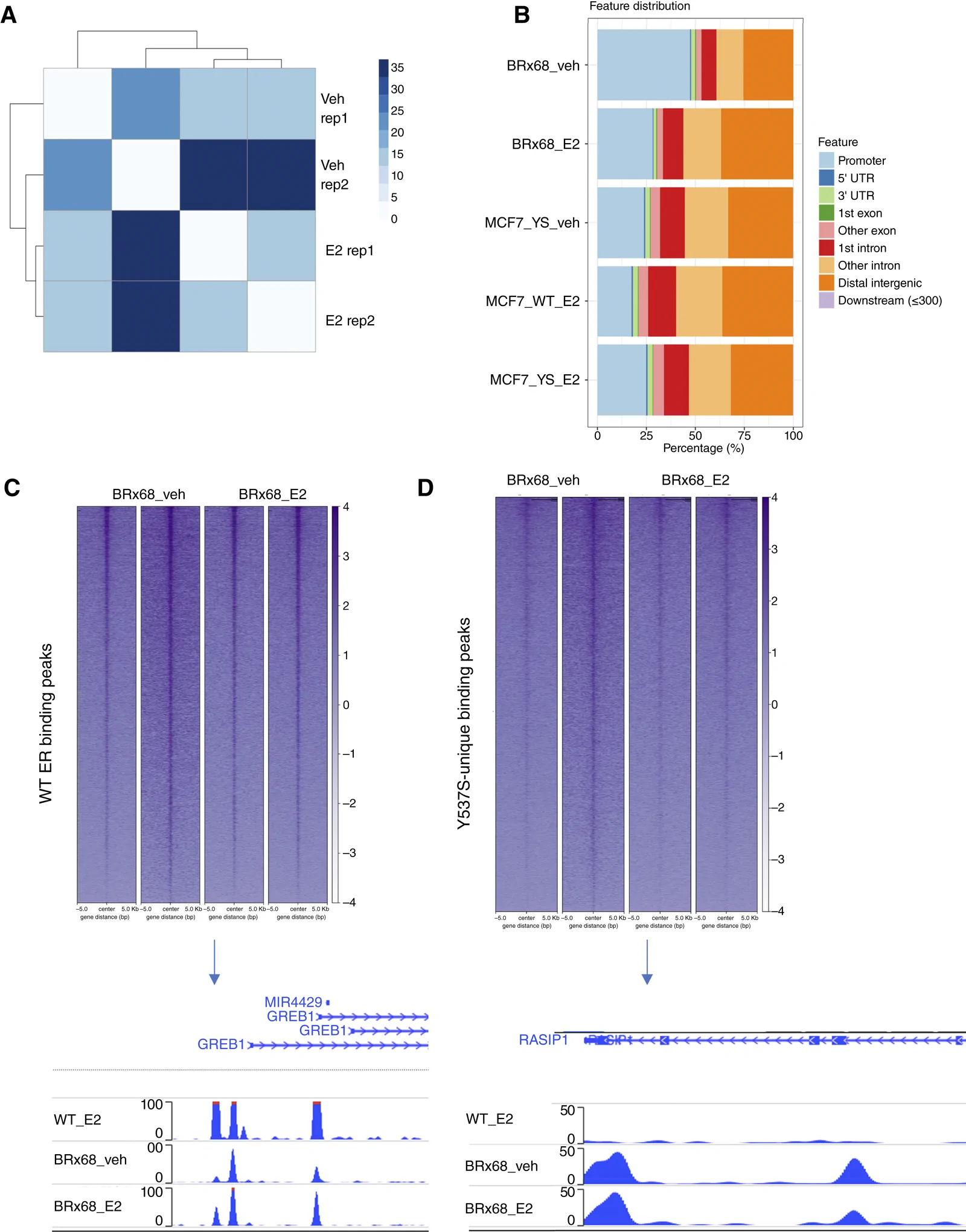
Feature distribution and heatmap of ER ChIP-seq data in the BRx68 CTC line. **A,** The heatmap shows pairwise similarity scores based on the percentage of overlapping statistically significant peaks between samples based on BRx68 ChIP-seq statistically significant called peaks. **B,** Feature distribution analysis of mutant ER compared with activated WT ER. Statistically significant called peaks (FDR less than 0.05 compared with input) of mutant ER from BRx68 in the presence and absence of E2 and MCF7 cells with doxycycline-inducible overexpression of mutant ER in the absence and presence of E2. The promoter is defined as 1 kb upstream and downstream of the TSS. **C** and **D,** Heatmaps of binding intensity of ER in BRx68 graphed against statistically significant called peaks from activated WT (**C**) or unique Y537S peaks (**D**). Unique Y537S peaks were identified by calling statistically significant peaks in MCF7 Y537S cells from Jeselsohn data and removing from the list peaks also called in MCF7 WT in the presence of E2. Underneath each graph is a representative region of each heatmap with bigwig files plotted using the Washington Epigenome Browser. UTR, untranslated region.

Several studies have performed ChIP-seq to analyze the binding profiles of hotspot mutant ERα using either genetically engineered cell lines via CRISPR knock-in (18, 19) or overexpression of inducible mutant ERα (17). Although these studies are important for studying the mutant receptor, there is still a lack of sufficient studies about the binding analysis of patient samples with *de novo ESR1* mutations. The BRx68 CTC line derived from a patient with luminal breast cancer was discovered to have a heterozygous Y537S *ESR1* mutation (15). We performed ERα ChIP-seq on the BRx68 cells in the presence and absence of the ERα ligand 17β-E2. We reasoned that as this was a heterozygous mutation, it would be important to study the ERα binding landscape in this CTC line both in the absence and presence of E2 in order to distinguish the roles of activating WT ERα (in the presence of E2) from a constitutively active Y537S mutant ERα (in the absence of E2). The top 1,000 variable binding regions of all the samples generated a heatmap that clusters together the two biological replicates of BRx68 in the presence and absence of E2 (Fig. 1A). Using a threshold of FDR value of less than 0.05 compared with its respective input, we identified 1,361 ERα binding peaks in the BRx68_veh samples and 1,864 peaks in the BRx68_E2 samples.

Next, we used ChIPseeker (29) to detect the genomic features of the peaks in BRx68 cells and compared them with the peaks detected in MCF7 cells using the ERα ChIP-seq data generated based on doxycycline-inducible expression of Y537S ERα (17). In the BRx68_veh condition, in which only the mutant ERα is active, a larger percentage of the peaks are found in the promoter regions, defined as 1 kb upstream and downstream of the TSS, whereas more peaks in distal intergenic regions were detected in the BRx68_E2 condition when WT ERα is also activated (Fig. 1B). This is intriguing because many ChIP-seq analyses of the WT ERα in ERα-positive breast cancer cell lines have shown that more peaks are typically found at distal enhancer sites than in promoters (44), as seen in the WT_E2 ChIP-seq data (Fig. 1B). Although the MCF7_Y537S_veh condition showed a higher percentage of binding in promoter regions than MCF7_WT_E2, there was no obvious difference between MCF7_Y537S_veh and MCF7_Y537S_E2 (Fig. 1B), which is different from BRx68. We then compared the ERα peaks in BRx68 cells against the common WT and Y537S ER peaks in MCF7 (Fig. 1C) or those specific to Y537S ER in MCF7 cells (Fig. 1D). Although BRx68_veh ERα shares 753 out of 1,361 peaks with both WT and Y537S ER in MCF7 cells, significantly fewer peaks overlap between BRx68_veh ERα and the Y537S ERα–unique regions, with only 124 out of 1,361 peaks shared, leaving 484 out of 1,361 peaks unique to BRx68_veh samples (Supplementary Fig. S1A). This indicates that between these two cell lines, the commonly shared Y537S ER binding regions are more likely to be traditional ER targets, whereas the unique Y537S ER targets are more likely to be cell type–specific.

### Identifying the intronic region of DPY19L1P1 as a unique Y537S ER binding site

**Figure 2.**
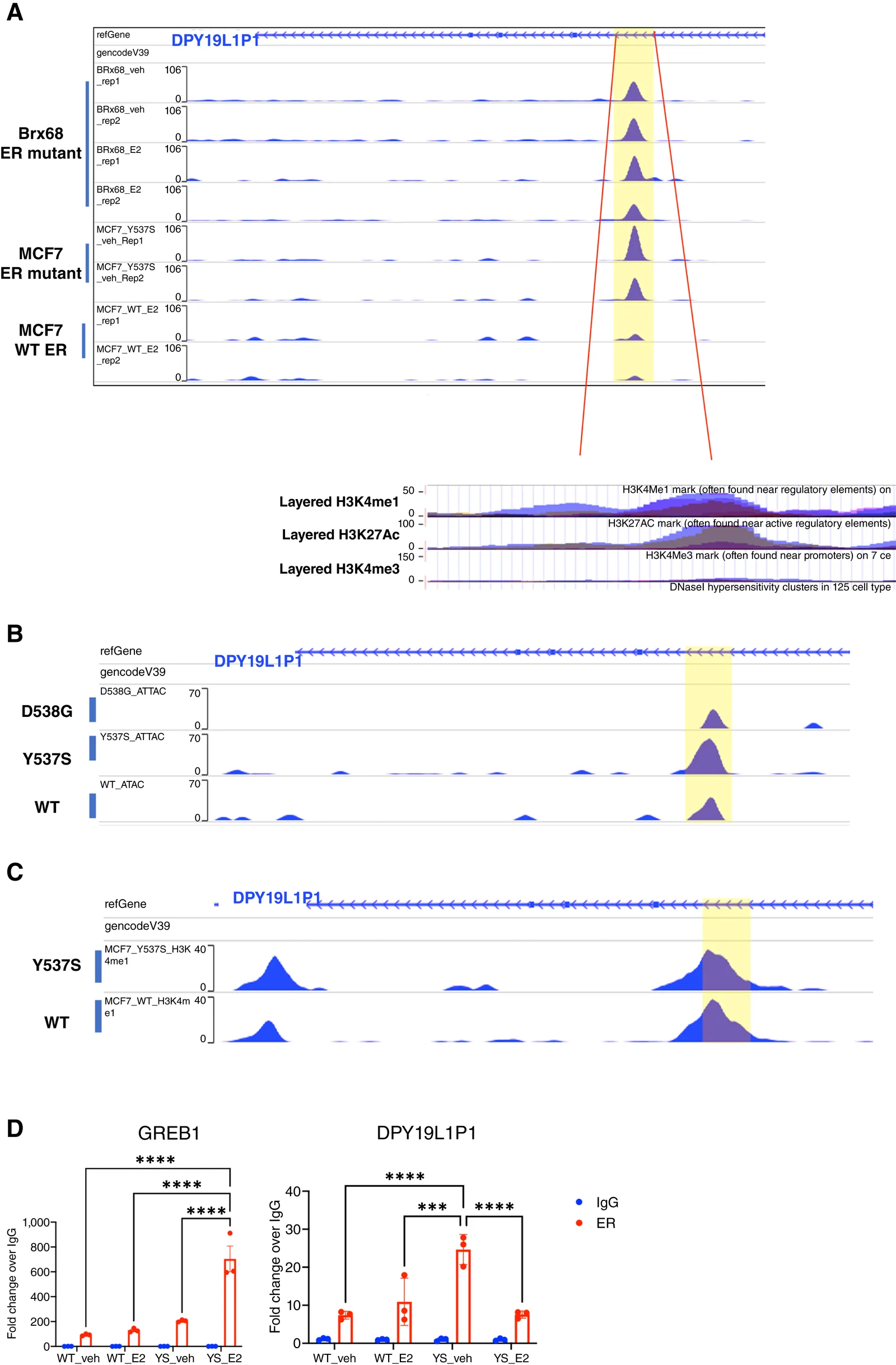
Identification of the DPY19L1P1 enhancer with a mutant ER-specific binding profile. **A,** Bigwig files from Jeselsohn and colleagues (17) and BRx68 ER ChIP-seq experiments displayed against the first intron of DPY19L1P1 using the Washington Epigenome Browser. Cumulative ENCODE data from H3K4me1, H3K27Ac, and H3K4me3 ChIP-seq experiments performed on seven different cell lines were listed below for the DPY19L1P1 intronic region. **B** and **C,** The bigwig files of ATAC-seq (**B**) and H3K4me1 CUT&Tag data (**C**) of WT, Y537S, and D538G MCF7 cells were shown for the DPY19L1P1 intronic region using the Washington Epigenome Browser. **D,** ER ChIP-qPCR analysis in WT and Y537S cells in the presence and absence of E2 with primers corresponding to the promoter of GREB1 (left) and the DPY19L1P1 region (right). Data were analyzed using two-way ANOVA with Tukey multiple comparison correction. ***, *P* < 0.001; ****, *P* < 0.0001.

Given the unique binding profiles of the BRx68_veh compared with the BRx68_E2 cells, consistent with the unique transcriptomes of mutant ERα variants established in the literature (17–20), we sought to determine the possibility of a unique mutant ERα binding region shared across different studies. Using the same pipeline employed for peak identification, we compared our ER ChIP-seq data from BRx68_veh with three previously published ChIP-seq datasets in activating mutant Y537S MCF7 cells (17, 19, 20) and identified a list of mutant ERα–unique peaks that are shared between at least one of the MCF7 studies and our BRx68 cells (Table 1). One region of interest was a 446 bp region in the first intron of a pseudogene called *DPY19L1P1* on chromosome 7 (Table 1). This region showed enriched binding of the mutant ER in BRx68 (Fig. 2A) and statistically significant increased binding of the mutant compared with WT in the previously published data (log_2_ fold change of more than 2; ref. 17). ChIP-seq data in ENCODE showed a strong H3K4me1 and H3K27Ac profile but a very minimal H3K4me3 mark in the DPY19L1P1 region (Fig. 2A), further supporting this region as an enhancer. This DPY19L1P1 region harbors a classic palindromic ERE site (AGGTCAcaaaGACCc) and a viable half-site in the antisense strand (caGGTCA), based on the TRANSFAC transcription factor motif database (Supplementary Fig. S1B). We next performed an ATAC-seq assay using a CRISPR-engineered Y537S ERα mutant in MCF7 cells compared with MCF7-WT cells, as well as another common ESR1 mutation, D538G, to determine whether there was open chromatin in the DPY19L1P1 region [Fig. 2B (top)]. Although Y537S, D538G, and WT cells all showed an open profile in the DPY19L1P1 region, there was a stronger intensity in the Y537S cells. To confirm the presence of specific H3K4me1 binding observed in the ENCODE data, we performed a CUT&Tag assay on both WT and Y537S cells using an antibody against the H3K4me1 mark and noted its enrichment in the region in both cells (Fig. 2C).

**Table 1.** Mutant ER–specific binding regions across multiple studies.

Study	Chr	Start	End	Closest gene	Region	H3K27Ac	H3K4me1/me3
Jeselsohn and colleagues (17)	10	124,133,825	124,134,396	PLEKHA1	Promoter	Strong	Weak/weak
Jeselsohn and colleagues (17)	12	30,907,815	30,908,345	LOC645485	Promoter	Strong	Weak/weak
Jeselsohn and colleagues (17)	7	8,301,637	8,302,054	ICA1	Promoter	Strong	Weak/weak
Jeselsohn and colleagues (17)	7	32,720,197	32,720,643	DPY19L1P1	Enhancer	Strong	Strong/weak
Jeselsohn and colleagues (17)	3	33,700,421	33,701,054	CLASP2	Enhancer	Strong	Strong/weak
Li and colleagues (20)	11	82,996,917	82,997,678	CCDC90B	Promoter	Strong	Weak/strong
Li and colleagues (20)	12	56,709,523	56,710,399	PAN2	Promoter	Strong	Weak/weak
Li and colleagues (20)	14	77,923,831	77,924,462	AHSA1	Promoter	Strong	Weak/weak
Li and colleagues (20)	16	30,933,634	30,934,905	FBXL19-AS1	Promoter	Strong	Weak/weak
Li and colleagues (20)	19	49,223,770	49,224,623	RASIP1	Enhancer	Strong	Weak/strong
Harrod and colleagues (19)	12	68,633,740	68,634,245	IL22	Enhancer	Weak	Strong/weak
Harrod and colleagues (19)	17	73,851,345	73,851,880	WBP2	Promoter	Strong	Weak/strong
Harrod and colleagues (19)	19	45,988,511	45,989,041	RTN2	Promoter	Strong	Weak/weak
Harrod and colleagues (19)	19	47,551,686	47,552,294	TMEM160	Promoter	Strong	Weak/weak
Harrod and colleagues (19)	19	48,833,247	48,833,681	EMP3	Enhancer?	Strong	Weak/weak

NOTE: A list of shared ER-binding peaks between genetically modified MCF7 with the Y537S ER mutation (17, 19, 20) and BRx68 is provided. The status of the enriched H3K27Ac and H3K4me1 histone marks from ENCODE for each region is listed. The determination of the strength of the peaks is based on the score from ENCODE, which is based on the average peak intensity of the histone mark at the respective region in the cell lines.

We next performed ChIP-qPCR to validate the binding of Y537S ER compared with WT ER, using primers against the DPY19L1P1 region and a well-known classic ER binding region in the promoter of the gene encoding *GREB1* as a positive control. WT and Y537S cells were cultured for 3 days in hormone-depleted serum and then treated for 24 hours with either 10 nmol/L of E2 or a vehicle control (ethanol). As expected based on previous reports (18, 45, 46), in the presence of E2, Y537S ERα showed statistically significant enriched binding to the promoter of *GREB1* compared with the WT (Fig. 2D). ChIP-qPCR against ERα in the DPY19L1P1 region also showed statistically significant enrichment in Y537S cells in the vehicle control compared with activated WT ER. Interestingly, we also noticed that when the Y537S cells were treated with E2 and thus activating the WT ERα in those cells, the ERα enrichment of the DPY19L1P1 decreased significantly compared with the vehicle condition (Fig. 2D).

### DPY19L1P1 KO disrupts MCF7 Y537S ER mutant–specific phenotypes

We knocked out this DPY19L1P1 enhancer region using CRISPR-Cas9 with sgRNAs targeting sites flanking the DPY19L1P1 region in both WT (D2) and Y537S (E5) MCF7 cells. Two clones of control KO were also generated for both WT and Y537S MCF7 cells at the same time using the same plasmids without the sgRNA sequence (WT_C10, WT_C11, Y537S_D4, Y537S_Z99). The KO of the DPY19L1P1 enhancer region was confirmed by PCR on genomic DNA using primers that were 200 bp upstream and downstream of the sgRNAs targeted regions, showing about a 700 bp shorter PCR product in WT_DPY_KO and Y537S_DPY_KO than the nonedited product (Supplementary Fig. S2A). We pooled the control cells in each genotype (WT_control and Y537S_control).

**Figure 3.**
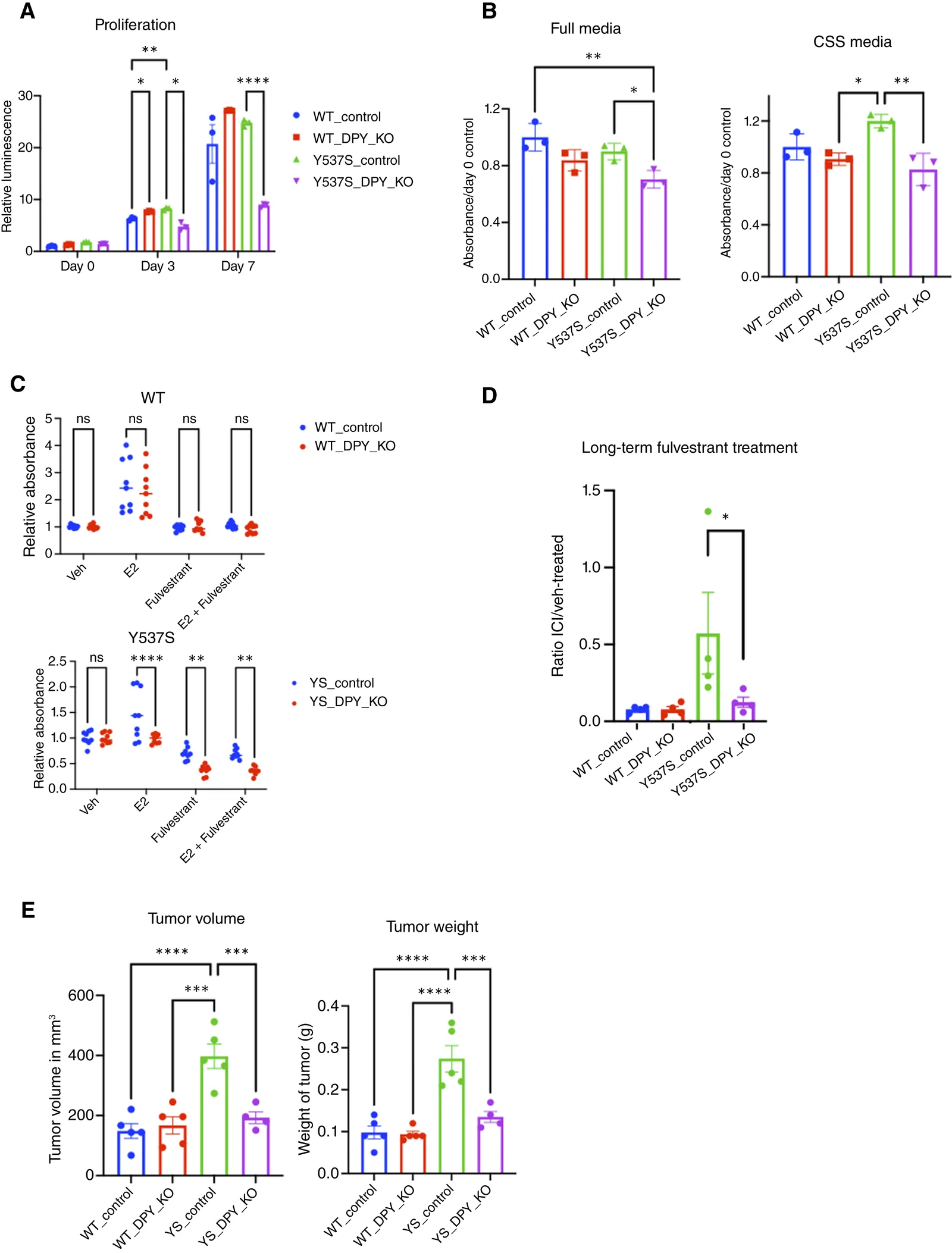
KO of DPY19L1P1 enhancer reduces cell proliferation and adhesion, increases sensitivity to Fulvestrant in Y537S MCF7 cells, and decreases tumorigenicity *in vivo*. **A,** Graph showing CellTiter-Glo2 reading of cells on days 0, 3, and 7 after culturing in complete serum (full media). Data were analyzed using two-way ANOVA and Tukey multiple comparison test. DPY_KO = DPY19L1P1 KO. **B,** Cell adhesion assay using crystal violet showing quantifications of cells grown in either full media (top) or CSS media (bottom). Data were analyzed using one-way ANOVA and Tukey multiple comparison test. **C,** MCF7 WT (top) and Y537S cells (bottom) grown in CSS and treated with either vehicle (DMSO/ethanol), 1 nmol/L of E2, 100 nmol/L of fulvestrant, or E2 + fulvestrant for 7 days. Values were normalized to the vehicle value for each graph. Data were analyzed using two-way ANOVA with Sidak multiple comparisons correction. **D,** Graph showing CellTiter-Glo2 results for fulvestrant treatment for 3 weeks. Fold changes were quantified from the initial seeding. Data were analyzed using Mann–Whitney unpaired *t* test comparing Y537S_control with Y537S_DPY_KO. **E,** Graphs showing the tumor volume (left) and weight (right) at 8 weeks after mammary fat pad injection of those cells in ovariectomized female mice. Data were analyzed using two-way ANOVA and unpaired *t* test. *, *P* < 0.05; **, *P* < 0.01; ***, *P* < 0.001; ****, *P* < 0.0001; ns, not statistically significant.

To evaluate the impact of DPY19L1P1 KO on mutant ER cell phenotypes, we first analyzed the influence on cell proliferation. CellTiter-Glo assay showed statistically significant attenuated growth of Y537S_DPY_KO compared with the Y537S_control cells in both the day 3 and 7 time points in full growth media condition (Fig. 3A). Interestingly, such attenuation was not detected between the WT_DPY_KO and WT_control conditions (Fig. 3A).

Next, we performed a cell adhesion assay in collagen-coated plates, as adhesion to the extracellular matrix could be important for metastatic outgrowth in the secondary organs (47). We performed the experiment in both complete serum (full media) and hormone-depleted CSS. In both conditions, the DPY_KO showed statistically significantly reduced adhesion compared with control in Y537S cells, but not in WT cells (Fig. 3B). These data indicate that deletion of the DPY19L1P1 enhancer region significantly reduced cellular proliferation and adhesion in the Y537S mutant MCF7 cells, but not in the WT MCF7 cells.

As endocrine therapy resistance is a hallmark of mutant ERα-bearing cells (12, 18), we sought to determine if deletion of the DPY19L1P1 region would enhance the sensitivity of these cells to a common SERD used to treat patients with breast cancer, fulvestrant (ICI). Cells with the DPY19L1P1 KO and their controls were grown for 3 days in CSS media and then seeded in 96-well plates and treated with either the vehicle or increasing concentrations of ICI (from 1 to 1,000 nmol/L) for 7 days, and cell proliferation was recorded by the CellTiter-Glo assay. As ICI targets ERα signaling through direct interactions with ER, we performed this experiment both in the presence and absence of E2. In WT cells, there were no significant differences between control and DPY_KO [Supplementary Fig. S2B (top)]. In Y537S cells, DPY_KO showed a statistically significant increase in sensitivity to 10 and 100 nmol/L of ICI compared with control in the absence of E2 [Supplementary Fig. S2B (bottom)].

We further validated the effect of ICI at the 100 nmol/L concentration in CSS media using a crystal violet method. Similar to the results using CellTiter-Glo, DPY_KO showed a statistically significant reduction in readings compared with the control in Y537S cells in both ICI treatment and E2 + ICI treatment (Fig. 3C), which was not observed in WT cells.

We next evaluated whether the sensitivity to ICI treatment in the Y537S_DPY_KO remained for a longer period of time. We performed a long-term treatment of 10 nmol/L ICI or vehicle for 3 weeks and calculated the proliferation rate from the starting time point. Both WT cells showed an average growth of less than 10% compared with vehicle-treated cells (Fig. 3D). This is consistent with literature suggesting that ICI is very potent at its normal concentrations in attenuating the growth of WT ERα-containing breast cancer cells (48). The Y537S_control cells showed an average of 50% growth in ICI treatment compared with vehicle treatment, whereas the Y537S_DPY_KO cells showed a statistically significant decrease in the ratio of proliferation compared with the Y537S_control cells, indicating that the enhanced sensitivity to ICI treatment acquired by the KO of the DPY19L1P1 region in Y537S cells is maintained for the long term.

Finally, we evaluated the impact of DPY19L1P1 KO on orthotopic tumor growth *in vivo*, particularly in low estrogen conditions to mimic the clinical scenario of low estrogen in patients with endocrine therapy resistance/mutant ESR1. MCF7 WT or Y537S control or DPY_KO cells were injected into the mammary fat pads of ovariectomized female NSG mice. Tumor volumes were measured by caliper over time, and tumor weight was recorded at 8 weeks. As expected, Y537S_control cells showed larger tumors compared with WT cells. Validating the *in vitro* data, DPY19L1P1 KO cells showed reduced tumor growth in both volume and weight specifically in Y537S but not in the WT background (Fig. 3E). In summary, these data showed that deletion of the DPY19L1P1 enhancer region disrupts MCF7 Y537S ERα mutant–specific phenotypes.

### *IGFBP3* and *CD44* are identified as potential downstream target genes of the DPY19L1P1 enhancer in Y537S cells

**Figure 4.**
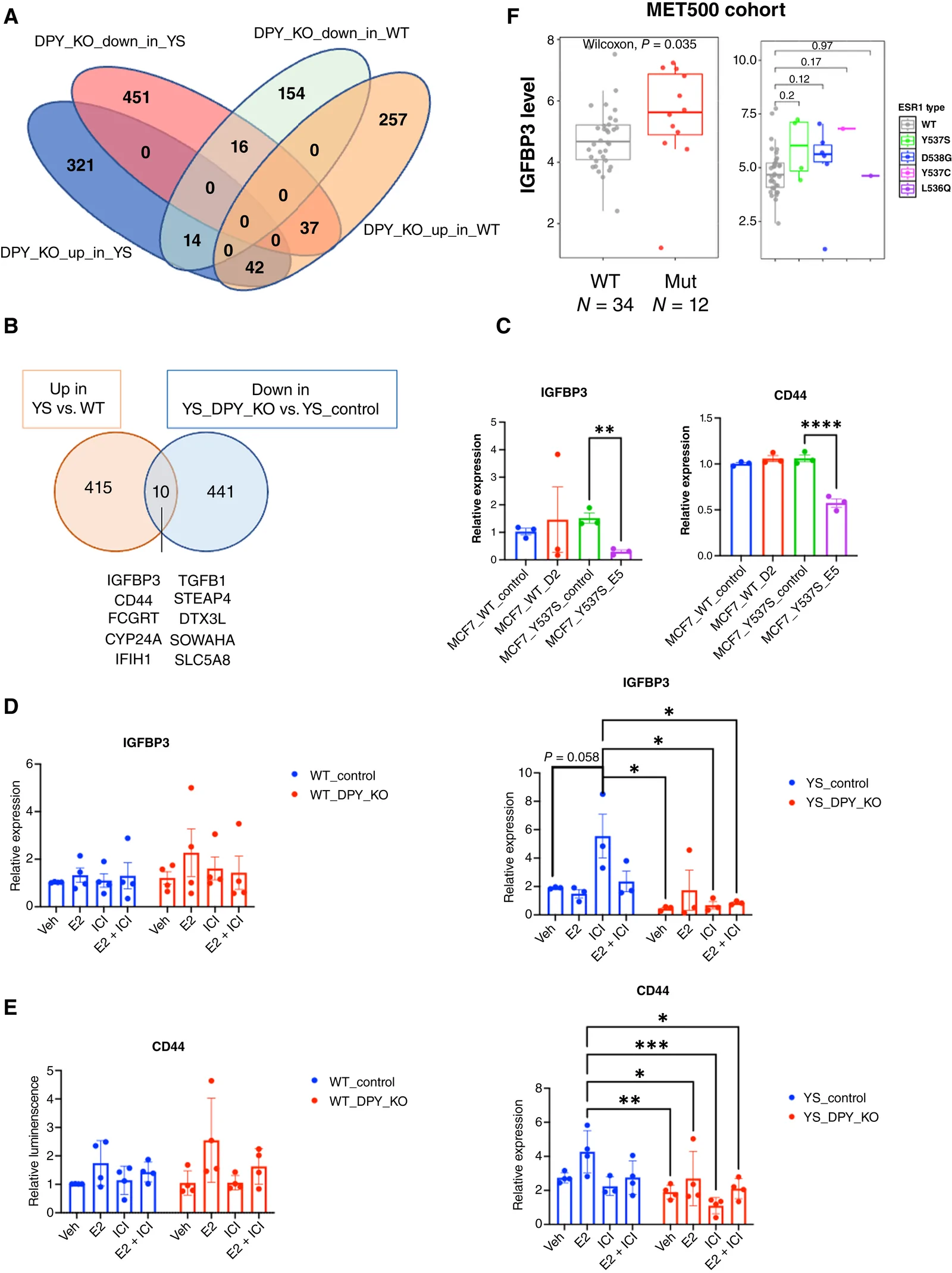
RNA-seq analysis identifies IGFBP3 and CD44 as potential downstream targets of DPY19L1P1 enhancer in Y537S cells. **A,** Venn diagram showing the intersection of differentially expressed genes derived from pairwise comparisons within specific genotypes upon DPY19L1P1 KO, based on a threshold of log_2_ fold change greater than 0 and FDR less than 0.05. **B,** Venn diagram showing the comparison made between differentially expressed genes from Y537S_veh compared with WT_E2 (left side) with genes downregulated in Y537S_DPY_KO compared with Y537S control (right side). **C,** qRT-PCR analysis of the relative RNA levels of IGFBP3 and CD44 in WT and Y537S cells with DPY19L1P1 KOs and their respective controls in full media. Data analysis was performed using one-way ANOVA with Dunnett multiple comparisons test. **D** and **E,** qRT-PCR analysis of the relative RNA levels of IGFBP3 (**D**) and CD44 (**E**) in WT (left side) and Y537S (right side) DPY_KO cells along with their controls grown in CSS media and treated with either vehicle (DMSO/ethanol), 1 nmol/L of E2, 10 nmol/L of fulvestrant (ICI), or E2 + ICI for 24 hours. Data were analyzed using two-way ANOVA with Tukey multiple comparisons test. **F,** Boxplot comparing IGFBP3 expression between ESR1 WT (*n* = 34) and ESR1-mutant (*n* = 12) tumors in the MET500 metastatic breast cancer cohort. Statistical significance was assessed using a two-tailed Wilcoxon rank-sum test. *, *P* < 0.05; **, *P* < 0.01; ***, *P* < 0.001; ****, *P* < 0.0001.

Enhancers are cis-regulatory elements that, while usually located within 1 Mb of the promoter, can also form long-distance interactions with the target genes (49). Although the enhancer–promoter interactions are mostly intrachromosomal, interchromosomal interactions also exist (50). To identify the possible gene targets of the DPY19L1P1 enhancer, we performed RNA-seq analysis using two replicates for WT_DPY_KO, Y537S_DPY_KO, and control clones. Differential expression analysis identified 503 downregulated (Supplementary Table S1) and 379 upregulated genes (Supplementary Table S2) by DPY19L1P1 KO in Y537S cells and 184 downregulated and 336 upregulated genes in WT cells. Among these, 451 and 321 are uniquely downregulated and upregulated genes in Y537S_DPY_KO cells, respectively (Supplementary Table S3; Fig. 4A).

We were interested in the genes upregulated in the Y537S compared with the WT cells that were also downregulated as a result of the DPY19L1P1 KO. Using published data of genetically edited Y537S MCF7 cells (18), we identified 425 Y537S-specific upregulated genes, among which 10 genes were also downregulated by deletion of the DPY19L1P1 region specifically in Y537S cells but not in WT cells. Among them were *IGFBP3*, found on chromosome 7 approximately 14 Mb away from the DPY19L1P1 region, and *CD44*, found on chromosome 11 (Fig. 4B). *IGFBP3* and *CD44* were upregulated in Y537S cells compared with WT and downregulated upon DPY19L1P1 deletion in Y537S cells, which was validated via qRT-PCR in both CSS media (Supplementary Fig. S3A) and full media (Fig. 4C).

We next analyzed these gene expression changes when treated with ICI by qRT-PCR. The positive control gene *GREB1* showed E2-mediated upregulation, which is suppressed by ICI (Supplementary Fig. S3B) as previously shown (17, 18). When the DPY19L1P1 enhancer is deleted in Y537S cells, there were statistically significant lower expressions of *IGFBP3* compared with the control treated with ICI (Fig. 4D) and *CD44* compared with the control treated with E2 (Fig. 4E). These data suggest that *IGFBP3* is more likely a potential downstream target of the DPY19L1P1 enhancer. To evaluate this finding in patient data, we analyzed RNA-seq data from the MET500 metastatic breast cancer cohort, in which there are 12 samples with ESR1 mutation compared with 34 WT ER. There is a statistically significant higher *IGFBP3* level in mutant compared with WT ER samples (Fig. 4F), corroborating the result found in cell lines.

### KO of IGFBP3 in Y537S cells reduces cell proliferation

IGFBP3 is involved mostly in transporting IGFs in the bloodstream to the transmembrane IGF type 1 receptor. Although IGFBP3 has been shown to display a proapoptotic phenotype (51–53), it has also been shown in recent studies to promote proliferation in different cancers (54). Given this data, we decided to explore the role of IGFBP3 in the Y537S cells.

**Figure 5.**
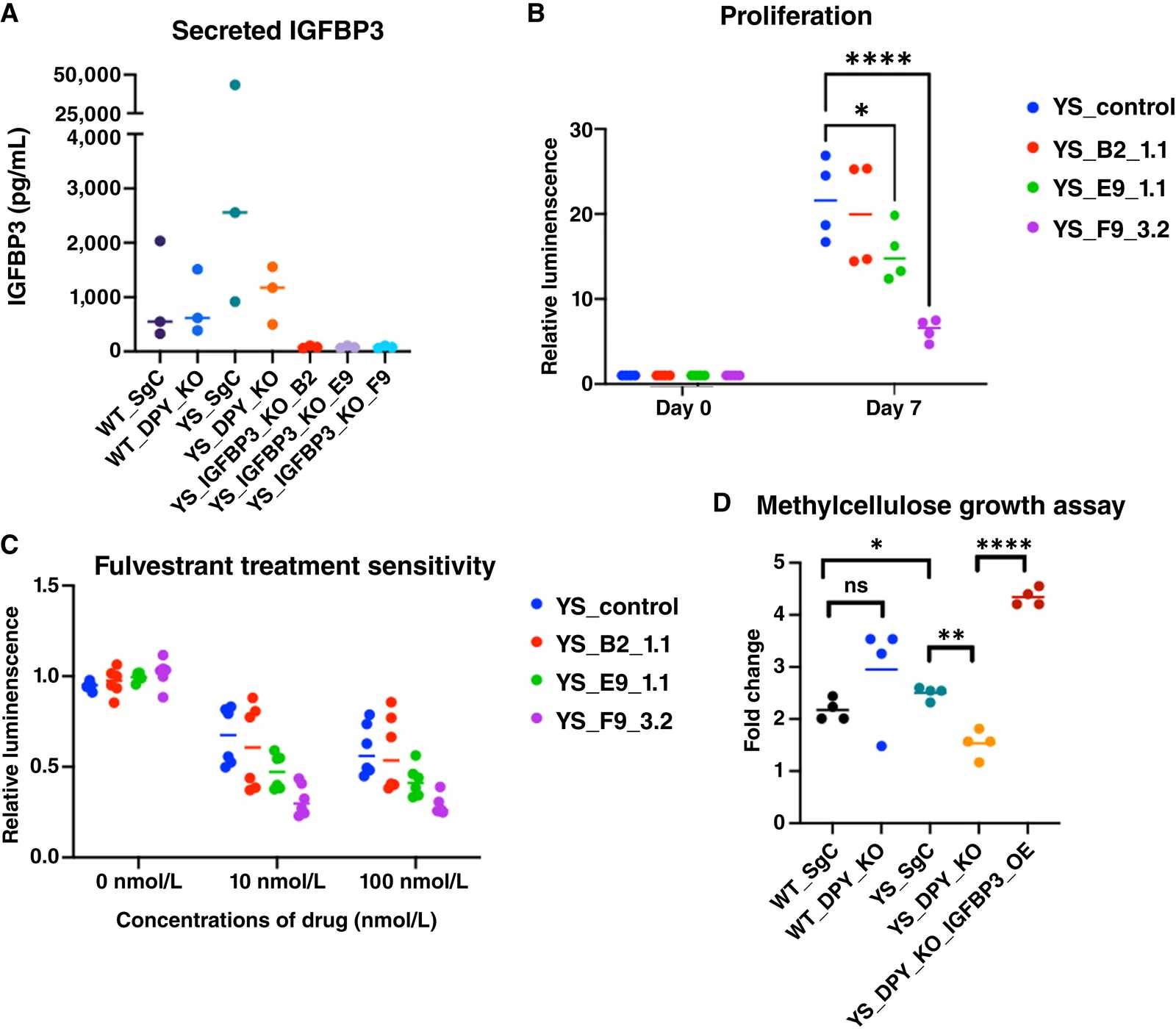
KO of IGFBP3 reduces cell proliferation in Y537S cells but not in response to fulvestrant. **A,** ELISA analysis of IGFBP3 levels in WT_SgC, WT_DPY_KO, YS_SgC, YS_DPY_KO, and different clones of Y537S cells transfected with two different sgRNAs targeting the IGFBP3 gene, sgRNAs 1.1 (B2 and E9) and 3.2 (F9), and a control clone (SgC). **B,** CellTiter-Glo2 results of IGFBP3 KO and control in Y537S cells grown in full media on day 0 or 7. Data were analyzed using two-way ANOVA with Dunnett multiple comparisons test. **C,** CellTiter-Glo2 results of Y537S control, IGFBP3 KO cells grown in CSS media and treated with either vehicle (DMSO/ethanol) or fulvestrant (ICI, either at 10 or 100 nmol/L) in the absence of E2 for 7 days. Values were normalized to the 0 nmol/L value for each graph. Data were analyzed using two-way ANOVA and Tukey multiple comparisons correction. **D,** Graph showing relative colony growth compared with day 1 in the methylcellulose growth assay in WT_SgC, WT_DPY_KO, YS_SgC, and YS_DPY_KO with empty control plasmid or with IGFBP3 overexpression (OE). Data were analyzed with Welch’s *T* test. *, *P* < 0.05; **, *P* < 0.01; ****, *P* < 0.001; ns, not statistically significant.

To validate the role of IGFBP3 in Y537S mutant MCF7 cells, we used CRISPR/Cas9 to KO *IGFBP3* and derive three clones. ELISA analysis validated a clear loss of IGFBP3 in the three clones (B2, E9, and F9; Fig. 5A). Two of these clones showed significantly reduced proliferation at day 7 compared with the control (Fig. 5B), whereas the third clone did not show reduced proliferation. However, the loss of IGFBP3 did not show a statistically significant difference in sensitivity to fulvestrant treatment (Fig. 5C), suggesting that there could be additional genes regulated by the DPY19L1P1 enhancer contributing to the resistance phenotype.

As the ER mutations are almost exclusively detected in metastatic breast cancers, we evaluated the effect of the loss of the DPY19L1P1 enhancer in cells growing in suspension to mimic the loss of anchorage effect during the metasetatic process (Fig. 5D). Quantification of the growth of cell colonies after 7 days in methylcellulose culture showed that Y537S SgC cells grow better than WT SgC. Interestingly, the loss of the DPY enhancer in WT did not lead to a statistically significant change. In contrast, the loss of the DPY enhancer in Y537S cells suppressed growth, whereas overexpression of IGFBP3 (Supplementary Fig. S4A) rescued this effect in DPY KO Y537S cells (Fig. 5D; Supplementary Fig. S4B), supporting an important role of IGFBP3 downstream of the DPY enhancer in the anchorage-independent growth of Y537S cells.

## Discussion

Extensive studies over the past decade, via sequencing of metastatic breast cancer tissues and CTCs, have identified a high prevalence of *ESR1* mutations in patients who have progressed on endocrine therapies (10, 12, 15–17). These mutations induce a conformational change in the ERα structure, allowing it to remain constitutively active even in the absence of its agonist, E2 (12, 18). Understanding the functions of mutant ERα is crucial for developing more effective targeted therapies and overcoming resistance. In our study, we compared the genomic ERα binding profiles between a CTC line heterozygous for Y537S ERα and genetically modified Y537S ERα in MCF7 cells. We discovered a novel Y537S ERα binding enhancer in the first intron of the DPY19L1P1 gene. Deleting this enhancer significantly reduced cell proliferation, adhesion, and anchorage-independent growth *in vitro*, reduced tumorigenicity *in vivo*, and enhanced the sensitivity of Y537S ERα-mutated cells to fulvestrant. We further identified *IGFBP3* as a functional downstream target of this enhancer and validated its role in mediating some of the phenotypes associated with Y537S ERα mutations.

Recent genomic and transcriptomic studies have been performed on breast cancer cell lines edited with the most common hotspot *ESR1* mutations to elucidate the mechanism by which the mutant ERα operates (17–19). Various ChIP-seq analyses have shown that although mutant ERα shares the majority of the WT ERα binding, they also have specific binding profiles. For example, Jeselsohn and colleagues (17) observed an increase in mutant ERα binding in the promoter regions of genes compared with the WT ERα, a trend we also observed with our ChIP-seq data in a CTC line carrying a *de novo* Y537S ERα mutation. This is interesting as WT ERα binds to ERE sites commonly found in distal intergenic regions. As most of the currently available mutant ERα binding profiles were derived from genetically engineered breast cancer cell lines, our ChIP-seq data from patient-derived CTCs with *de novo ESR1* mutations are of unique value in confirming the insights from established breast cancer cell lines, however with the caveat of only one such patient-derived sample.

Despite the known cell type–specific ERα binding effect, we identified a novel mutant ERα binding enhancer that is shared between the BRx68 CTC line harboring a heterozygous Y537S mutation and MCF7 that were edited to include the Y537S *ESR1* mutation. Located at the first intron of a pseudogene called DPY19L1P1, this region showed enhancer-like properties with enriched peaks for H3K4me1 and H3K27Ac in ENCODE profiles. ATAC-seq data on MCF7 cells showed the region to be open in cells regardless of the genotype but had the greatest intensity in the Y537S cells. Furthermore, a CUT&Tag assay performed in MCF7 WT and Y537S cells confirmed the enrichment of H3K4me1 at this particular region in both cell lines. Functional validation of this region revealed that its deletion in MCF7 cells reduces proliferation and adhesion and increases sensitivity to fulvestrant when the cells harbor the ERα Y537S mutation. To our best knowledge, this is the first functionally validated Y537S mutant ERα-enriched enhancer.

RNA-seq analysis revealed two genes of interest, *IGFBP3* and *CD44*, that are upregulated by Y537S ER and downregulated upon the enhancer deletion. Importantly, IGFBP3 KO curtailed the growth of Y537S cells, and overexpression of IGFBP3 in DPY-KO rescued the reduced anchorage-independent growth in Y537S cells. However, knocking out IGFBP3 in Y537S cells did not significantly sensitize the cells to fulvestrant treatment. These data suggest that IGFBP3 contributes partially to the phenotypes observed in DPY19L1P1 enhancer deletion but that additional genes regulated by this enhancer are also important to the phenotypes examined. Given the well-known role of CD44 in adhesion (55) and tumorigenesis (56), it is possible that *CD44* could be one such gene contributing to these phenotypes.

The role of IGFBP3 in cancer is a subject of controversy. In some studies, it has been identified as a proapoptotic, antitumor gene (51, 57). For example, GRP78 was identified as an interacting partner of IGFBP3, and IGFBP3 overexpression in endocrine therapy–resistant breast cancer cells led to a disruption of the GRP78–caspase-7 complex and induced apoptosis (51). Patients who expressed IGFBP3 and had low expression of GRP78 experienced poor prognosis (58), suggesting that IGFBP3’s role in apoptosis is largely based on its interaction with GRP78. On the other hand, some studies have shown that IGFBP3 is associated with poor cancer prognosis (59) and promotes tumorigenesis (58). A previous publication showed that nuclear IGFBP3 is associated with tumor progression in breast cancer (54), which was attributed to the activation of the epidermal growth factor receptor, EGFR, and sphingosine kinase (SphK). SphK1 has also been linked with ERα signaling (60) and tumorigenesis (61). It will be interesting to evaluate the nuclear role of IGFBP3 on mutant ERα activity in future studies.

Several questions remain to be addressed in future studies. Our RNA-seq approach yielded a list of genes that may be regulated by the DPY19L1P1 enhancer. Here, we focused on validating IGFBP3 due to its relative proximity to the enhancer site and its association with poor prognosis in patients with breast cancer (59, 62). However, the physical interaction of the DPY19L1P1 region with the promoter of its target genes is yet to be validated using chromatin capture technologies, which would yield important insights into the functions of this mutant ERα–enriched enhancer region. In addition, DPY19L1P1 is an expressed pseudogene, which could act through a noncoding RNA-related mechanism regulating downstream gene expression. This possibility is not evaluated here but may be worth further investigation in future studies.

Moreover, there are other recently reported insights on *ESR1* mutant cells that are not directly regulated by mutant ERα. These include additional non-ERα targets related to cell–cell adhesion (20), elevated basal cytokeratins and associated enhanced immune cell recruitment (63), and FOXA1 reprogramming that leads to enhanced retinoid X receptor binding (64). It will also be interesting to evaluate whether the identified enhancer region plays any role in those regulations or the effect of ER–androgen receptor interactions, as also recently reported (65).

In summary, our study discovered that a region in the first intron of the DPY19L1P1 gene displayed characteristics of an enhancer, and the deletion of this region attenuated growth in both anchorage-dependent and independent conditions, reduced cell adhesion, and increased sensitivity to a common endocrine therapy, fulvestrant, specifically in Y537S mutant ERα cells. The identification of this mutant ERα–enriched enhancer, which demonstrates a clear functional phenotype, could be leveraged to improve targeted therapies for patients with the Y537S ERα mutation.

## Supplementary Material

Supplemental Figure 1 Motif of the DPY19L1P1 enhancer region and validation of the KO.

Supplemental Figure 2 Validation of the KO of DPY19L1P1 enhancer and its effect

Supplemental Figure 3 RT-qPCR analysis of IGFBP3, CD44, and GREB1

Supplemental Figure 4 Overexpression of IGFBP3 in YS DPY_KO cells

Supplemental Table 1 Downregulated genes in Y537S DPY_KO compared to Y537S Control cells

Supplemental Table 2 Upregulated genes in Y537S DPY_KO compared to Y537S Control cells

Supplemental Table 3 Uniquely downregulated genes in Y537S DPY_KO compared to Y537S Control cells but not in WT cells

## Data Availability

Sequencing data generated in this study have been deposited in the Gene Expression Omnibus under accession numbers GSE333218, GSE333366, and GSE333367. Other data generated in this study are available from the corresponding author upon request.
