# Supplementary material for: Identification and Validation of the ESR1 Y537S Mutant–Enriched Enhancer and Its Downstream Target IGFBP3
Source: Cancer Res Commun. 2026 Oct 14;6(10):2395–411. doi: 10.1158/2767-9764.CRC-26-0366 (PMC13662453; doi:10.1158/2767-9764.CRC-26-0366)
Supplement: Supplementary file 1 [file crc-26-0366_supplemental_figure_1_suppsf1.pdf]

Supplemental Figure 1

A

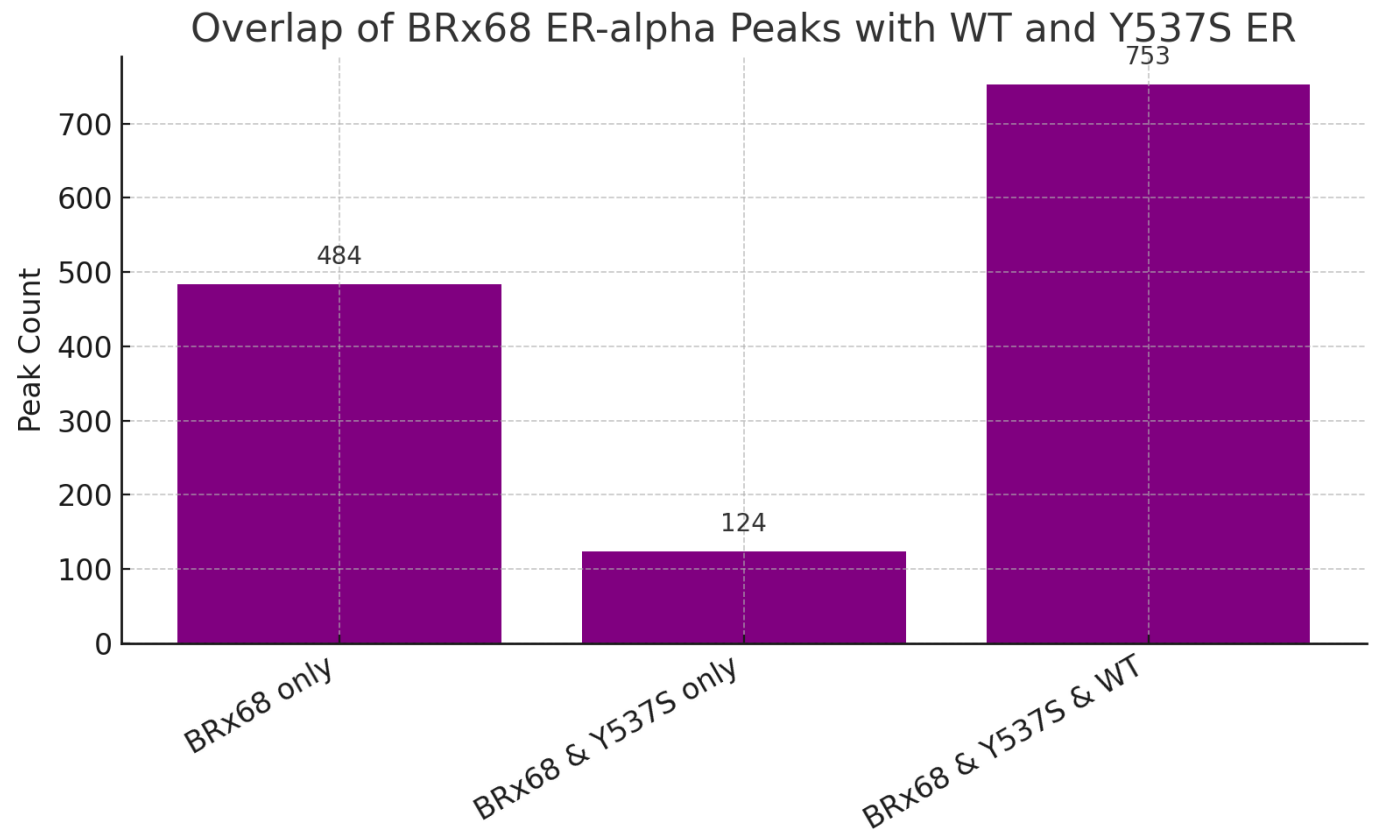

B

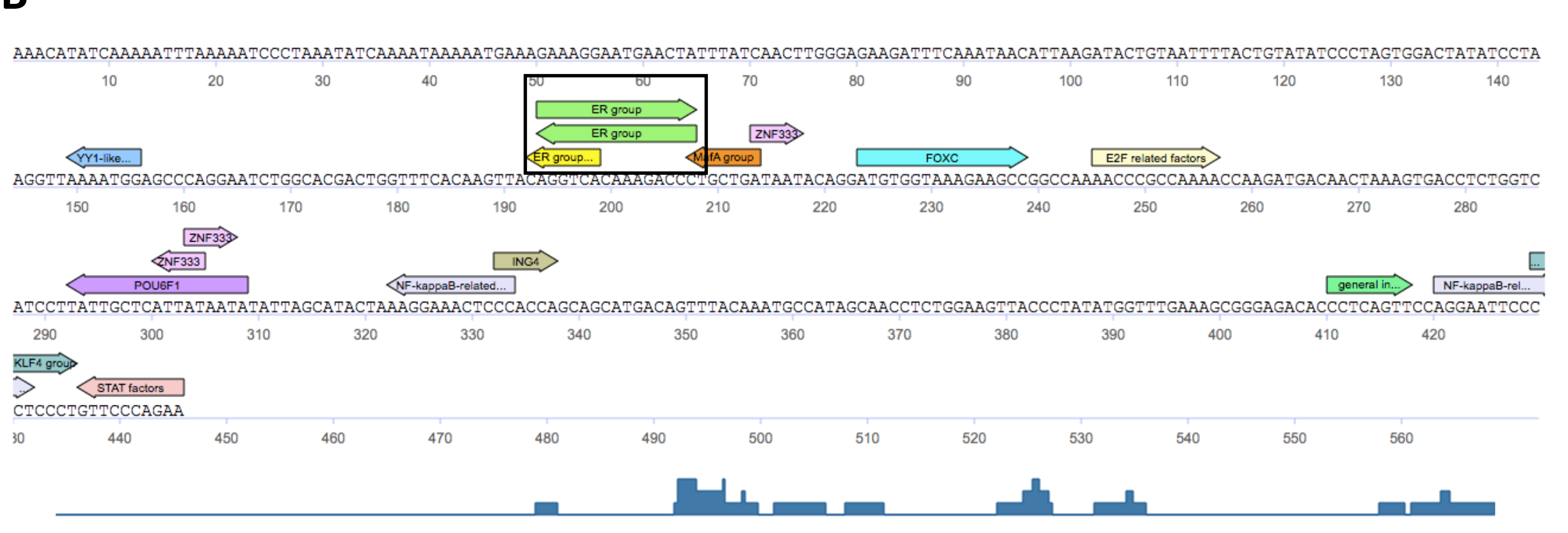

Supplemental Figure 1. Motif of the DPY19L1P1 enhancer region and validation of the KO. **(A)** Bar plot showing intersection of ERα binding peaks across BRx68_veh, WT ER, and Y537S ER samples. Visualizing the distribution and overlap of estrogen receptor alpha (ERα) ChIP-seq peaks among BRx68_veh, WT_ER, and Y537S_ER datasets (the latter two from Jesselsohn et al 2018).

(B) Transcription factor binding site predictions using JASPAR for the *DPY19L1P1* region. A palindromic ERE and an antisense half-ERE motif are identified in the *DPY19L1P1* region (black box).
