# Supplementary material for: Identification and Validation of the ESR1 Y537S Mutant–Enriched Enhancer and Its Downstream Target IGFBP3
Source: Cancer Res Commun. 2026 Oct 14;6(10):2395–411. doi: 10.1158/2767-9764.CRC-26-0366 (PMC13662453; doi:10.1158/2767-9764.CRC-26-0366)
Supplement: Supplementary file 2 [file crc-26-0366_supplemental_figure_2_suppsf2.pdf]

Supplemental Figure 2

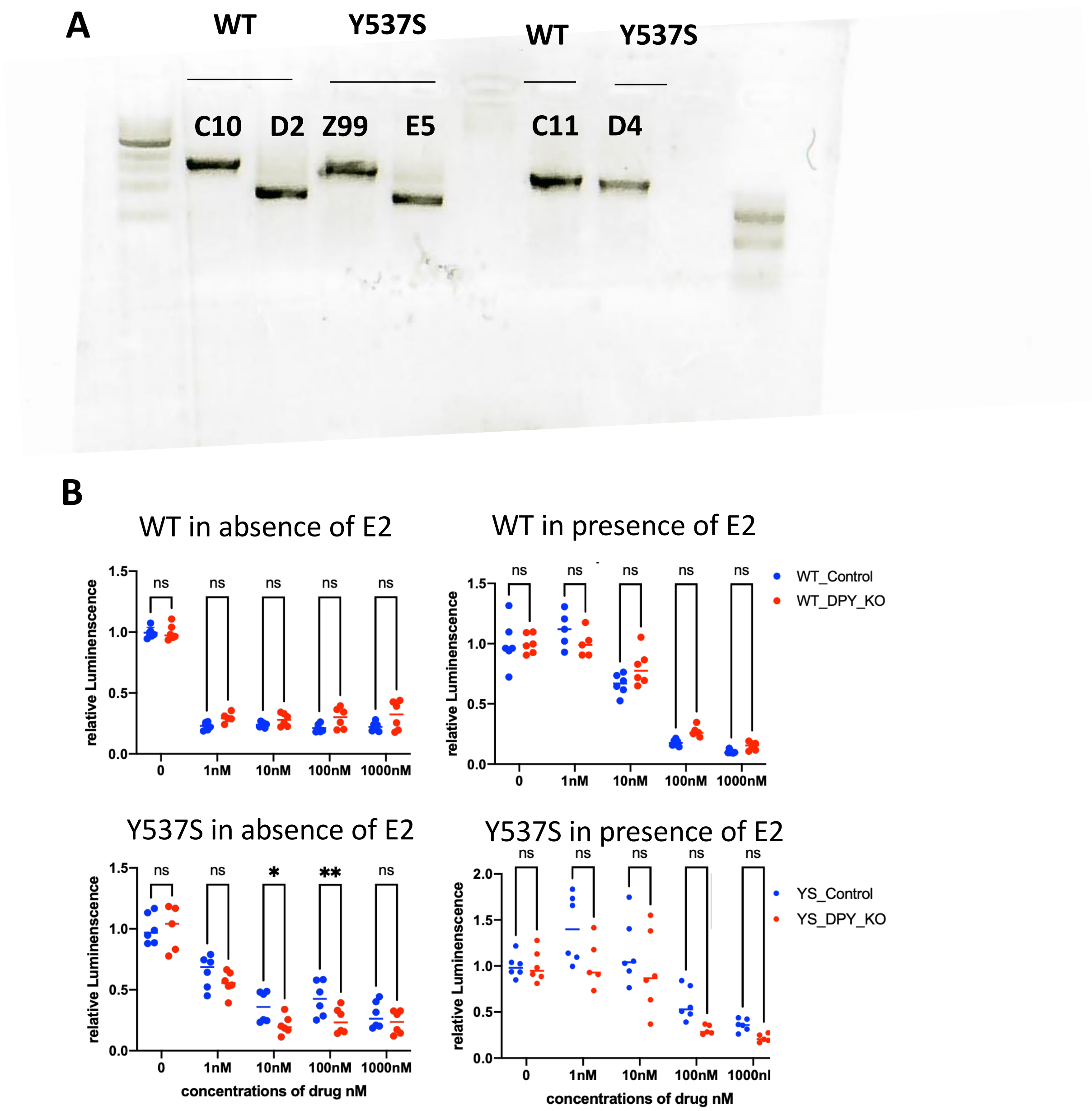

Supplemental Figure 2. Validation of the KO of DPY19L1P1 enhancer and its effect.

(A) Gel images of PCR product using primers targeting 200bp upstream and downstream of the expected DPY19L1P1 knockout region on genomic DNA extracted from the established single cell clones from WT and Y537S MCF7 cells. 1 Kb ladder (left) and 100bp ladder (right) were run alongside the samples.

(B) Graphs showing CellTiter-Glo2 results for MCF7 WT (top) and Y537S cells (bottom) grown in CSS and treated with either vehicle (DMSO/Ethanol) or fulvestrant at a concentration ranging from 1nM to 1000nM, without (left) or with (right) 1nM of E2, for 7 days. Values were normalized to the 0 nM value for each graph. Experiment was performed 6 times. Data was analyzed using 2-way ANOVA with Sidak's multiple comparison correction. * P< 0.05 ** P <0.01, *** P < 0.001, **** P<0.0001; YS = Y537S.
