# Supplementary material for: Identification and Validation of the ESR1 Y537S Mutant–Enriched Enhancer and Its Downstream Target IGFBP3
Source: Cancer Res Commun. 2026 Oct 14;6(10):2395–411. doi: 10.1158/2767-9764.CRC-26-0366 (PMC13662453; doi:10.1158/2767-9764.CRC-26-0366)
Supplement: Supplementary file 3 [file crc-26-0366_supplemental_figure_3_suppsf3.pdf]

Supplemental Figure 3

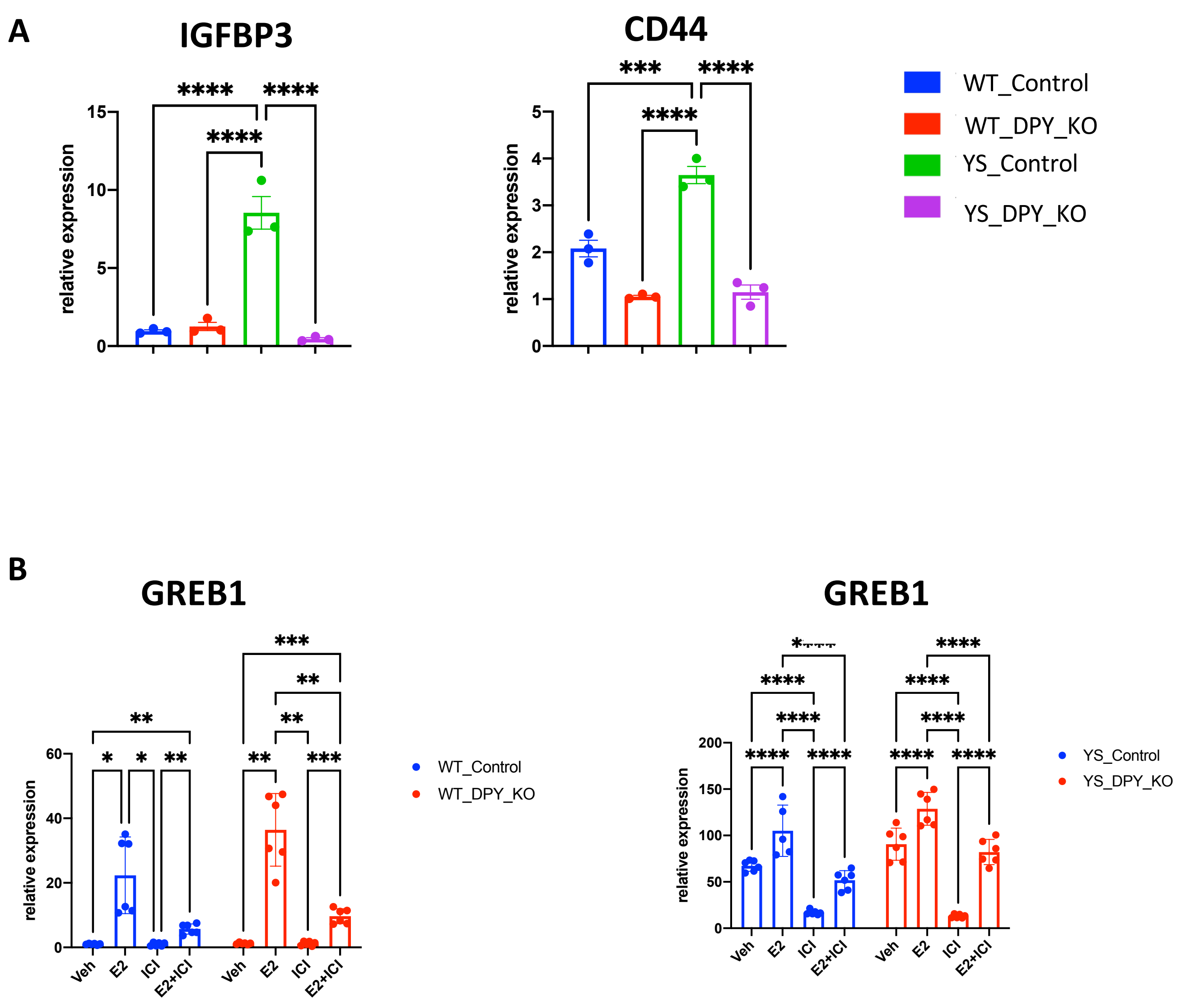

Supplemental Figure 3. RT-qPCR analysis of IGFBP3, CD44, and GREB1.
(A) Bar graphs showing qPCR analysis of relative RNA expression level of IGFBP3 and CD44 in WT and Y537S cells with DPY19L1P1 KOs and their respective control in CSS media. Data analysis was performed using one-way ANOVA analysis with Dunnett's multiple comparison test. * P< 0.05 ** P <0.01, *** P < 0.001, **** P<0.0001 N=3.
(B) WT (left side) and Y537S (right side) DPY19L1P1 KO cells along with their controls were grown in CSS media for 3 days and then seeded and treated with either vehicle (DMSO/Ethanol), 1nM of E2, 10nM of fulvestrant (ICI), or E2 + ICI for 24 hours. RNA was then processed, cDNA was synthesized, and qPCR was performed using primers against classic ER target gene GREB1. DPY_KO = DPY19L1P1 Knockout.
