# Supplementary material for: Identification and Validation of the ESR1 Y537S Mutant–Enriched Enhancer and Its Downstream Target IGFBP3
Source: Cancer Res Commun. 2026 Oct 14;6(10):2395–411. doi: 10.1158/2767-9764.CRC-26-0366 (PMC13662453; doi:10.1158/2767-9764.CRC-26-0366)
Supplement: Supplementary file 4 [file crc-26-0366_supplemental_figure_4_suppsf4.pdf]

Supplemental Figure 4

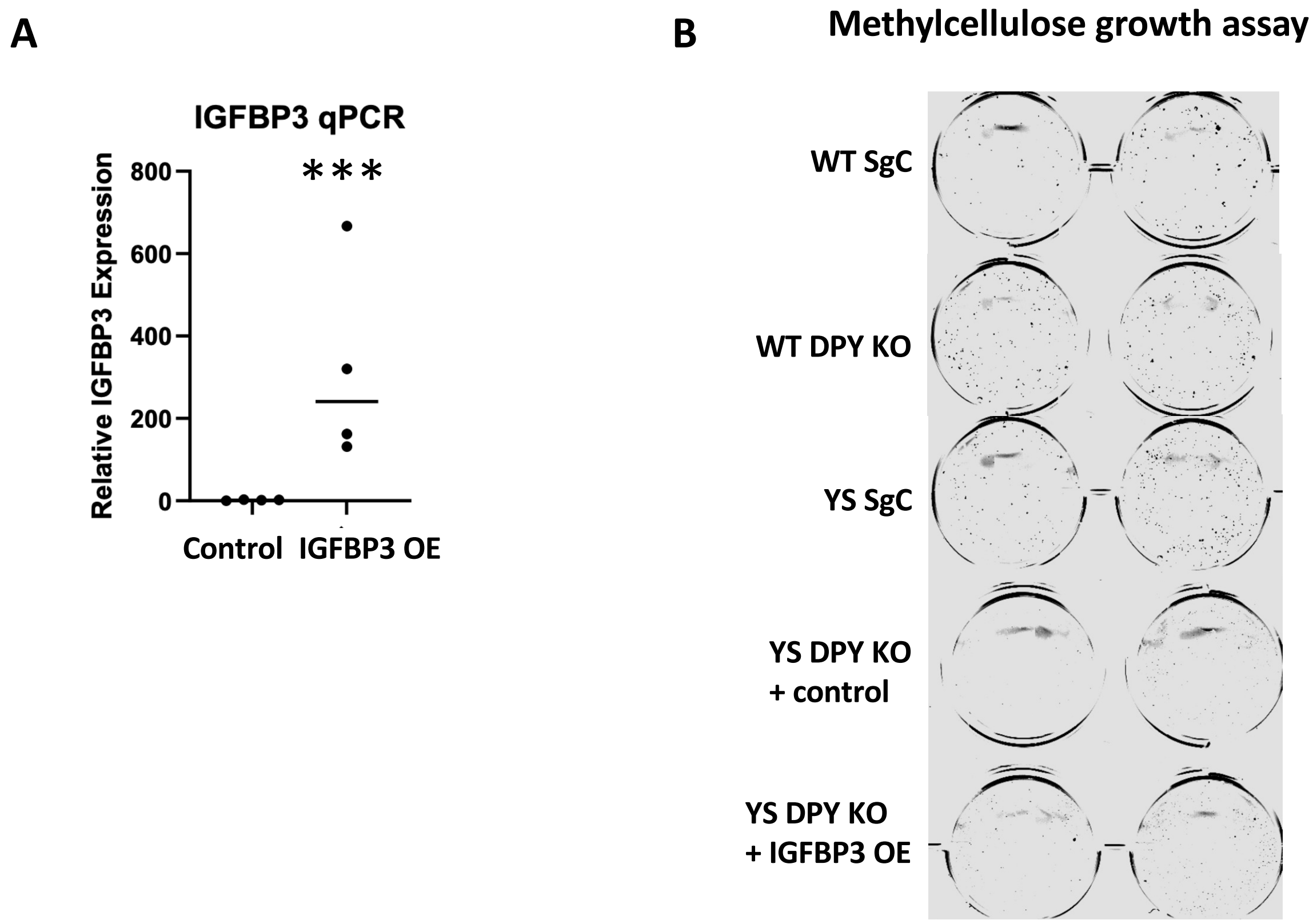

Supplemental Figure 4. Overexpression of IGFBP3 in YS DPY_KO cells.
(A) qPCR analysis of relative RNA expression level of IGFBP3 Y537S cells with DPY19L1P1 KO cells that transduced with empty vector control or overexpression of IGFBP3. *** $P < 0.001$, N=4.
(B) Representative images of the methylcellulose colony growth assay (2 wells each as technical replicates in one biological experiment) at day 7. GFP signal has been changed to black color for quantification.
